# Human-aided dispersal and population bottlenecks facilitate parasitism escape in the most invasive mosquito species

**DOI:** 10.1093/pnasnexus/pgae175

**Published:** 2024-04-30

**Authors:** Maxime Girard, Edwige Martin, Laurent Vallon, Van Tran Van, Camille Da Silva Carvalho, Justine Sack, Zélia Bontemps, Julie Balteneck, Florence Colin, Pénélope Duval, Simon Malassigné, Ian Hennessee, Lucrecia Vizcaino, Yamila Romer, Nsa Dada, Khan Ly Huynh Kim, Trang Huynh Thi Thuy, Christophe Bellet, Gregory Lambert, Fara Nantenaina Raharimalala, Natapong Jupatanakul, Clement Goubert, Matthieu Boulesteix, Patrick Mavingui, Emmanuel Desouhant, Patricia Luis, Rémy Cazabet, Anne-Emmanuelle Hay, Claire Valiente Moro, Guillaume Minard

**Affiliations:** University of Lyon, Université Claude Bernard Lyon 1, CNRS, INRAe, VetAgro Sup, UMR Ecologie Microbienne, F-69622 Villeurbanne, France; University of Lyon, Université Claude Bernard Lyon 1, CNRS, INRAe, VetAgro Sup, UMR Ecologie Microbienne, F-69622 Villeurbanne, France; University of Lyon, Université Claude Bernard Lyon 1, CNRS, INRAe, VetAgro Sup, UMR Ecologie Microbienne, F-69622 Villeurbanne, France; University of Lyon, Université Claude Bernard Lyon 1, CNRS, INRAe, VetAgro Sup, UMR Ecologie Microbienne, F-69622 Villeurbanne, France; University of Lyon, Université Claude Bernard Lyon 1, CNRS, INRAe, VetAgro Sup, UMR Ecologie Microbienne, F-69622 Villeurbanne, France; University of Lyon, Université Claude Bernard Lyon 1, CNRS, INRAe, VetAgro Sup, UMR Ecologie Microbienne, F-69622 Villeurbanne, France; University of Lyon, Université Claude Bernard Lyon 1, CNRS, INRAe, VetAgro Sup, UMR Ecologie Microbienne, F-69622 Villeurbanne, France; Department of Medical Biochemistry and Microbiology, Science for Life Laboratories, Uppsala University, 752 37 Uppsala, Sweden; University of Lyon, Université Claude Bernard Lyon 1, CNRS, INRAe, VetAgro Sup, UMR Ecologie Microbienne, F-69622 Villeurbanne, France; Université de Lyon, INSA Lyon, Université Claude Bernard Lyon 1, CNRS, UMR 5240 MAP, Microbiologie, Adaptation, Pathogénie, F-69622 Villeurbanne, France; University of Lyon, Université Claude Bernard Lyon 1, CNRS, INRAe, VetAgro Sup, UMR Ecologie Microbienne, F-69622 Villeurbanne, France; University of Lyon, Université Claude Bernard Lyon 1, CNRS, INRAe, VetAgro Sup, UMR Ecologie Microbienne, F-69622 Villeurbanne, France; University of Lyon, Université Claude Bernard Lyon 1, CNRS, INRAe, VetAgro Sup, UMR Ecologie Microbienne, F-69622 Villeurbanne, France; Rollins School of Public Health, Emory University, Atlanta, GA 30322, USA; Entomology Branch, Division of Parasitic Diseases and Malaria, Center for Global Health, US Centers for Disease Control and Prevention, Atlanta, GA 30333, USA; Emory College of Arts and Science, Emory University, Atlanta, GA 30322, USA; Centre de Recherche pour la lutte contre les Maladies Infectieuses Tropicales/Tropicales Infectious Diseases Research Centre, Université d’Abomey-Calavi, Cotonou, Benin; School of Life Sciences, Arizona State University, Tempe, AZ 85281, USA; Department of Medical Entomology and Zoonotics, Pasteur Institute, 722700 Ho Chi Minh City, Vietnam; Department of Medical Entomology and Zoonotics, Pasteur Institute, 722700 Ho Chi Minh City, Vietnam; Entente Interdépartementale Rhône-Alpes pour la Démoustication, 73310 Chindrieux, France; Entente Interdépartementale de Démoustication du Littoral Méditerranéen, 34000 Montpellier, France; Pasteur Institute, Antananarivo, Madagascar; National Center for Genetic Engineering and Biotechnology (BIOTEC), 12120 Pathum Thani, Thailand; Canadian Centre for Computational Genomics, McGill Genome Centre, Human Genetics, McGill University, QC H3A0G1 Montreal, Canada; Laboratoire de Biométrie et de Biologie Evolutive, Université de Lyon, Université Claude Bernard Lyon 1, UMR CNRS 5558, VetAgro Sup, F-69622 Villeurbanne, France; UMR PIMIT, Processus Infectieux en Milieu Insulaire Tropical, CNRS 9192, INSERM U1187, IRD 249, Université de La Réunion, 97490 Sainte-Clotilde, La Réunion, France; Laboratoire de Biométrie et de Biologie Evolutive, Université de Lyon, Université Claude Bernard Lyon 1, UMR CNRS 5558, VetAgro Sup, F-69622 Villeurbanne, France; University of Lyon, Université Claude Bernard Lyon 1, CNRS, INRAe, VetAgro Sup, UMR Ecologie Microbienne, F-69622 Villeurbanne, France; Univ de Lyon, CNRS, Université Lyon 1, LIRIS, UMR5205, F-69622 Villeurbanne, France; University of Lyon, Université Claude Bernard Lyon 1, CNRS, INRAe, VetAgro Sup, UMR Ecologie Microbienne, F-69622 Villeurbanne, France; University of Lyon, Université Claude Bernard Lyon 1, CNRS, INRAe, VetAgro Sup, UMR Ecologie Microbienne, F-69622 Villeurbanne, France; University of Lyon, Université Claude Bernard Lyon 1, CNRS, INRAe, VetAgro Sup, UMR Ecologie Microbienne, F-69622 Villeurbanne, France

**Keywords:** parasite, invasion, transportation, biogeography, microbiota

## Abstract

During biological invasion process, species encounter new environments and partially escape some ecological constraints they faced in their native range, while they face new ones. The Asian tiger mosquito *Aedes albopictus* is one of the most iconic invasive species introduced in every inhabited continent due to international trade. It has also been shown to be infected by a prevalent yet disregarded microbial entomoparasite *Ascogregarina taiwanensis*. In this study, we aimed at deciphering the factors that shape the global dynamics of *A. taiwanensis* infection in natural *A. albopictus* populations. We showed that *A. albopictus* populations are highly colonized by several parasite genotypes but recently introduced ones are escaping it. We further performed experiments based on the invasion process to explain such pattern. To that end, we hypothesized that (i) mosquito passive dispersal (i.e. human-aided egg transportation) may affect the parasite infectiveness, (ii) founder effects (i.e. population establishment by a small number of mosquitoes) may influence the parasite dynamics, and (iii) unparasitized mosquitoes are more prompt to found new populations through active flight dispersal. The two first hypotheses were supported as we showed that parasite infection decreases over time when dry eggs are stored and that experimental increase in mosquitoes’ density improves the parasite horizontal transmission to larvae. Surprisingly, parasitized mosquitoes tend to be more active than their unparasitized relatives. Finally, this study highlights the importance of global trade as a driver of biological invasion of the most invasive arthropod vector species.

**Significance Statement**

Global trade expansion has facilitated the introduction of invasive species such as the Asian tiger mosquito *Aedes albopictus*. Eventually, invasive species might escape their natural enemies and this phenomenon exemplifies their invasion success. In this study, we combined field observations and laboratory experiments to decipher the ecological consequences of the invasion process on the interaction dynamics between *A. albopictus* and its most prevalent natural parasite *Ascogregarina taiwanensis*. We observed a decrease in parasitism in recently introduced populations and provide experimental evidence to explain how human-aided mosquito transportation and mosquito population bottlenecks were a burden for the parasite.

## Introduction

Despite repeated calls on actions to prevent drastic consequences of global change, our society is now facing many ecological challenges that could profoundly affect its future (1). The cumulative effects of biological invasion and human activities are a threat to biodiversity due to change in global species distributions but also species loss and drift in community composition related to parasite transmission or competitive exclusion (2–7). Introduced species tend to raise health, agricultural, goods, and services costs that weaken the global economy (8, 9). Finally, some invasive species are considered as a threat to human health due to their pathogenicity or their ability to transmit pathogens (10). Currently, 37% of the 66 most-studied invasive species are considered as a threat, while uncertainties remain on others (11). During biological invasion, invaders may be accompanied by parasites that may originate from their native range or an intermediate location (12). Cointroduction of invasive species with their parasites can lead to native species infection also referred as host shifting when the infection concerns different host species or spillover when the infection concerns different host populations (13). Invasive species may also acquire the parasite from native species. Finally, following introduction, invasive species may also escape the parasite colonizing them in their native or intermediate areas (14). Those events could contribute to exacerbate the performance and invasion success of recently introduced host species.

Both stochastic and selective pressures may lead to parasite release (15). Subsampling of individuals in the native or intermediate area during the invasion process may lead to uninfected newly formed populations. This is particularly true when the parasite prevalence is low within the source population (16, 17). Furthermore, unsuitable conditions during the invasion path or colonization process may also lead to differential loss of the parasite due to e.g. unsuitable transportation conditions, higher host resistance at the invasion front and nonpermissive environmental conditions (18). Other studies suggest that demographic changes may also alter the parasite transmission among individuals at the invasion front (19, 20). Indeed, new populations are often founded by a small number of individuals that may be less prompt to exchange parasite due to lower horizontal transmissions and parasite virulence (21). In some cases, lower interindividual transmission due to low population densities is counter balanced by a plastic change in host permissiveness toward parasites named the density-dependent prophylaxis (DDP) (22). In such cases, the immune system is relaxed in conditions of low host densities and reciprocally upregulated in conditions of high host densities. It is therefore expected that parasitism release is more likely to be exemplified if reduction in population densities occurs in absence of DDP. Finally, parasitism release may be the result of a parasite-driven counter selection on the host propensity to disperse. As an example, the parasite *Ophryocystis elektroscirrha* decreases flight performance of the Monarch butterfly *Danaus plexippus* reducing the chance of infected individuals to migrate (23).

The Asian tiger mosquito *Aedes albopictus* is one of the most invasive species and poses threats for human health (24) due to its ability to replicate and transmit at least 19 viruses such as Chikungunya, Dengue, or Zika, as well as human and animal filarial nematodes (25, 26). Originating from South and East Asia, its distribution has rapidly increased all over the world since 1980s (27). Many studies pointed out the strong influence of human-aided transportation on intermediate and long-distance dispersal of desiccated mosquito eggs and, in a lesser extent, adults (27–33). Actually, *A. albopictus* mosquitoes are often transported as desiccated eggs on attractive containers (e.g. used tires and lucky bamboo) (28, 34–39). Eggs hatch whenever the containers get flooded after being introduced in a new area. Under optimal conditions, previous studies have shown that *A. albopictus* eggs can survive a month-long desiccation period (40). However, due to harsh transport conditions and population subsampling, the demography of recently introduced mosquito populations is often small (41, 42). Such founder effects involve genetic drift, genetic bottlenecks, and reductions in population size at the invasion front; however, these populations were shown to experience rapid growth in subsequent years (42–46). The final step of the invasion process is the local spread of mosquitoes and is both mediated either via flight or via passive transportations again (42, 47).

*Aedes albopictus* populations are highly infected by an Apicomplexan gregarine parasite belonging to the *Ascogregarina* genus (48). Various species of *Ascogregarina* are colonizing mosquitoes with host specificity (49). *Ascogregarina taiwanensis* is specifically associated with *A. albopictus* even though rare spillover events toward nonhost mosquito species were reported (50–54). Its biological cycle is synchronized with the mosquito development (55). Oocysts released outside the eggshell are ingested by first instar larvae in water containers and replicate into developing individuals until adults release them. This parasite can be horizontally transmitted either when adults die, defecate or emerge within larval habitats. *Ascogregarina taiwanensis* may have strong ecological consequences for its mosquito host although it is considered as a weak parasite (56). It was shown to have detrimental effects on mosquito traits such as development and survivorship at high density or when food is limited (57–59). A previous study conducted in Florida (USA) suggested that *A. albopictus* may escape *A. taiwanensis* at the invasion front (60). The authors demonstrated a drastically reduced parasite prevalence in recently introduced mosquito populations (<3 years). To reinforce those preliminary observations, we have conducted a field study in mainland France showing a similar trend of parasitism release at the invasion front. To estimate whether stochastic selection of uninfected individuals was likely to occur, we have also tested older populations in the native range and intermediate locations. We showed that the parasite is one of the most prevalent and dominant members of the mosquito microbiota in settled populations among contrasted locations across the world. Considering that stochastic selection of uninfected individuals was unlikely, we have thus conducted hypothesis driven experiments to test factors that may explain the observed release from the parasite pressure during biological invasion.

Our experiments have been designed to answer three questions based on the mosquito invasion process: (i) Does long-distance dissemination through human-aided egg transportation limit the parasite maintenance? (ii) Does the decrease in host density following introduction affect the parasite dissemination probability in absence of DDP? and (iii) Does the parasite limit mosquito local active dissemination through flight? To answer these questions, we first tested the effect of desiccation on the parasite maintenance to reflect conditions occurring during long-distance dissemination. Then, we tried to reproduce the decrease in mosquito populations reflecting founder effect through two different experiments to assess the impact of both adult and larvae densities on the parasite infection success. Finally, the impact of the parasitism on mosquito flight has been assessed through mosquito movement counting. Combined with previously published data, our results support that population density decrease following founding events and human-aided transportation contribute both to parasitism escape. Furthermore, our results suggest that the parasite de novo colonizes the mosquito with high densities 2 years after introduction.

## Material and methods

### Rearing parasitized and unparasitized mosquito lines

A total of ∼100 larvae were collected in 2017 in Villeurbanne (N: 45°46′18990″ E: 4°53′24615″) and Pierre-Bénite (N: 45°42′11534″ E: 4°49′28743″) in France. This population named AealbVB was grown into larger populations (∼5,000 individuals) for 15 generations in a Biosafety level 2 insectary at 28 °C, with a relative humidity of 80% and a day/night cycle of 18 h/6 h. Once the eggs were laid, they were immediately placed into water and ∼100 adults individuals were crushed and spread to make sure that the parasite was loaded. The water was never changed until the individuals molt into the second instar since individuals get infected during the first instar. This protocol pursued for each generation. Uninfected individuals originate from the same initial population. To ensure that the parasite did not infect them, several actions were performed to shunt the infection path. First, the eggs were not immersed immediately but water was removed and eggs were kept dry at least 7 days before being flooded again to avoid direct transmission of this parasite when it is released by adults in water. Then, no contact was permitted between infected individuals and the water in which larvae were reared to avoid any direct transmission from feces or adult bodies. Finally, as the infection occurs during the first stage of larval development, the water was changed during the first instar to reduce the transmission probability. The absence of infection in the unparasitized population and presence of infection in the parasitized population was regularly checked by crushing adult individuals in water and observing the mixture at a magnification of × 400 on an optical microscope (Leica).

### Sample collection and prevalence estimation

Live mosquito samples were collected with aspirators, net, and BG-traps in 7 countries and 17 sites. They were identified using morphological characteristics (61), stored in 70% ethanol during transportation and frozen until use. Information related to site, country, year of collection, sex, climate, GPS coordinates and the population age (i.e. time between population introduction and sample collections) are reported in Table S1. This last information was obtained from local entomological surveillance services in France (EID and EIRAD), as well as from published reports in Italy (62), Spain (63), and United States (64). The date of introduction in Madagascar is relatively uncertain and was estimated to be older than 1904 (65). Populations from Thailand and Vietnam were considered as natives. DNA was extracted from surface sterilized mosquito whole bodies and PCR diagnostic were performed to determine the parasite prevalence (see Supplementary material). To complete those datasets, supplemental data of *A. taiwanensis* presence–absence were extracted from previously published studies corresponding to mosquitoes collected in the United States (57, 60, 66, 67) either from the manuscript or from graph by using the ImageJ software v.1.54f (68). Data are available in Table S2.

### Metabarcoding of eukaryotic and prokaryotic microbial communities

Using DNA extracted from surface sterilized mosquitoes, prokaryotic and eukaryotic communities within entire individuals were characterized through high throughput sequencing of hypervariable regions from the 16S rDNA and 18S rDNA genes, respectively. The V5–V6 region of 16S rDNA gene was amplified using the primers 784F (5′-AGG ATT AGA TAC CCT GGT A-3′) and 1061R (5′-CRR CAC GAG CTG ACG AC-3′). The V1–V2 region of the 18S rDNA was amplified using the primers Euk82F (5′ GAA ACT GCG AAT GGC TC 3′) and Euk516R (5′ ACC AGA CTT GCC CTC C 3′). The details concerning PCR cycles are presented in Supplementary material. The PCR products were then sent to Biofidal sequencing company for purification and 2 × 300 bp Miseq sequencing (Illumina). A total of 12,836,921 and 14,248,382 reads were obtained from 16S and 18S rDNA, respectively, and were demultiplexed using the Mothur pipeline (69) that is described in Supplementary material. After removing singletons and subsampling, 5,886 and 5,264 OTUs were retrieved, respectively, for 16S rDNA and 18S rDNA. Contaminant OTUs were removed if their proportion in samples were not at least 10-fold in comparison with controls. Finally, after quality control, data were homogenized by sampling 1,000 and 500 reads for 16S rDNA and 18S rDNA, respectively. The data have finally been converted into relative abundance; hereafter, defined as the proportion of reads associated to each OTU in comparison to the total number of reads per sample. Since relative abundances are of compositional nature, we have randomly selected 7 samples from each sex and group of microbial similarities for which we conducted 16S and 18S rDNA qPCR to convert relative abundances into absolute abundances, i.e. number of copies associated with the given OTU per mosquito sample (see more details in Supplementary material). Miseq sequences have been deposited on Zenodo under the project number 8252320 (https://zenodo.org/record/8252320).

### Testing the ability of parasite maintenance during passive long-distance transportation on desiccated eggs

As previously described, desiccated mosquito eggs can be transported by human transportations during weeks. However, we do not know if such transportation could affect the parasite. Since *A. albopictus* tends to prefer dark container (70), we let infected mosquitoes lay eggs on water containers coated with a dark green blotting paper and preserved them in the dark under optimal conditions (i.e. 28 °C with a relative humidity of 80%). After respectively 35 h, 1 week and 1 month, 100 eggs were harvested from the blotting paper and placed in a water container to hatch. Larvae were reared at 28 °C and fed 1/3 yeast (Biovers) and 2/3 fish food and was provided ad libitum to the larvae. The adults that emerged from those containers were collected. The number of parasites colonizing the adult mosquitoes was estimated with qPCR on 10 males and females per time point and replicate. Primers and protocol for *A. taiwanensis*-specific qPCR were developed for this study to determine the absolute abundance of the parasite (i.e. number of the parasite 18S copy per mosquito) and are detailed in Supplementary material. The experiment was reproduced 5 times. This experiment was repeated 5 times on five different blotting papers.

### Testing the influence of mosquito densities on interindividual transmission

Water containers were maintained with 50 mL of sterile water in cages containing either 100 (low-density) or 3,000 (high-density) infected adult individuals during a week to allow them to release oocysts in water. The experiment was replicated 5 times for each condition (i.e. 5 donor mosquito cages were used for each tested mosquito density). Eggs from an unparasitized population were hatched in sterile water and 50 larvae were added to the container. Larvae were reared in similar conditions to the previously described experiment. Adult males and females were collected at emergence and their parasite density was measured with qPCR using 4 to 10 individuals per sex and replicate.

### Testing DDP

To test whether the interindividual transmission could be related to the density of larvae into the breeding sites, we hatched >1,000 larvae in containers to let them get infected with the parasite released from their parents. After 2 days, we then harvested them and grew them in new containers filled with distilled water at different densities (either 20, 50, or 100 larvae per 100 mL of water). This protocol was replicated 5 times for each condition. Larvae were reared in a container place on a shelve providing shadow. When adults emerged, they were collected and parasites quantification was performed with qPCR (5 males and 5 females per replicate and larval density). For technical reasons, highest density modality could not be performed for the fifth replicate (i.e. poor hatching rate for this replicate). Larval rearing conditions were similar to those in the previous experiment.

### Testing the influence of the parasite on active local mosquito dispersal through flight

Absence of the parasite in recently introduced mosquito populations might be the result of a poor propensity of infected mosquitoes to be active and fly. To test such hypothesis, parasitized and unparasitized adults were collected 2 weeks after their emergence and each individual was placed in a 25 mm Ø activity tube containing a cotton soaked with a 10% sucrose solution and sealed with cotton. The tube was placed in a LAM25 Locomotor Activity Monitor system (Trikinetics). The system was crossed by nine infrared beams that register an activity signal whenever the mosquito crosses the beams. Mosquitoes were left for 15 min to rest before their activity was measured. The system was placed on a shelve that provides shadow to the mosquitoes since they tend to prefer such habitats in nature (71). The total activity measured with LAM systems can be mostly attributed to flight since walk distance do not exceed 20% of mosquitoes activity (72). Two experiments each involving 16 parasitized males, 16 parasitized females, 16 unparasitized males, and 16 unparasitized females were conducted for 24 h in a BSL2 insectary with 18 h/6 h day/night and 28 °C. Whenever individuals died before the end of the registration, they were removed from the final dataset.

### Statistical and data analysis

#### Variations of *A. taiwanensis* prevalence in field populations

Statistical analyses were performed with the R software v.4.3.1 (73). The proportion of parasitized mosquitoes (defined hereafter as parasite prevalence) has been modeled for field populations as a response variable using a binomial distribution and a probit link function. The explanatory variables tested were the sex and/or the mosquitoes collection site (defined hereafter as the mosquito origin), using the full dataset or a subsampled dataset for each sex to study the impact of factors without taking into account statistical interactions. The influence of each factor was tested with a type II Wald χ^2^ test (*lme4* and *car* packages). We also correlated average parasite prevalence in collection sites with the number of years after the mosquito introduction as well as difference in prevalence between sites with the geographical distances separating them using the Spearman rank index with a correlation test and the Moran's I autocorrelation coefficient, respectively (*stat* and *ape* packages). Geographical distances between mosquito populations were calculated based on ellipsoid distance (*enmSdm*, package). Correlation analyses were repeated with data corresponding to previously published datasets from samples collected in the United States at different locations and time points after introduction.

#### Microbial communities associated with field populations

Microbiota diversity was estimated using the number of OTUs per individual sample after rarefaction for richness, Shannon index for alpha diversity, and Bray–Curtis dissimilarity index for beta diversity (*vegan,* package). Richness and alpha diversities were modeled using a Poisson distribution and a log link function or a Gaussian distribution, respectively, and the impact of the sex and population origin were used as fixed factors. The fixed factors were tested with a type II ANOVA or Wald χ^2^ test (*lme4* and *car,* packages). The influence of sex and population origin on microbial Bray–Curtis dissimilarity was tested using permutational multivariate analysis (*vegan,* package). A similar analysis was used to compare the influence of the years after introduction and climates for each sex on the microbiota. Moreover, a mantel correlation test was used to compare the Beta diversity distances to geographical distances. Relative abundances of the 20 most dominant prokaryotic and eukaryotic OTUs were represented as a heatmap (*Complexheatmap,* package). The phylogenetic analysis of *A. taiwanensis* OTUs (based on an alignment of the most abundant sequences in each OTU) was performed with Seaview and the Muscle algorithm using other members of the Apicomplexa phylum. Phylogenetic trees were then generated with a Maximum Likelihood method, an HKY evolutionary model and 100 bootstraps (performed with Seaview and represented with Figtree). To identify the microorganisms which relative abundances covaried with the population age (years after introduction of *A. albopictus* on each sampling sites), a constrained analysis of principal coordinates (CAP) was performed (*vegan,* packages). The microbiota abundance correlation networks were performed with a homemade *python* code and visualized with the *pyvis* library. Briefly, the 200 most frequently retrieved OTUs for each community (prokaryotic and eukaryotic) were selected and their paired nonparametric correlations were tested. If the Spearman correlation (*R*s) value was >0.25 (positive correlation) or <−0.25 (negative correlation), a linked was added between the two pairs. An automatic positioning algorithm was used based on mass-spring models. In this model, the links play a spring role which tend to bring the OTUs together, while the OTUs play a role of opposite mass charges that are constantly repelled from each other. The algorithm used iterations to minimize the energy of the system. Individual *A. taiwanensis* OTUs that vary according to mosquito population age were correlated with this last variable using nonlinear Spearman correlations. Since relative abundances may induce bias due to their compositional nature, we have repeated CAP and correlation analyses with the selected samples for which we have performed qPCR.

#### Parasite prevalence, abundance, intensity, and flight activity in controlled experiments

For the founder effect and desiccation experiments, the abundance and the intensity (i.e. abundance of the parasite in infected individuals) of *A. taiwanensis* per mosquito were modeled with mixed models with a Gaussian distribution using mosquito sex, density, or desiccation as fixed factors and the experimental replicates as random variables (*lme4*, package). Fixed factors were tested with an ANOVA or a type II Wald χ^2^ test. Post hoc Tukey-HSD tests were used to estimate pairwise differences (*emmeans*, package). Prevalence was also tested for the experiment in which enough parasitized and unparasitized mosquitoes were detected (at least 5 events of each). It was analyzed with mixed model using a binomial distribution with a logistic link function with the mosquito sex, desiccation as fixed factors and tested with a type II Wald χ^2^ test. Flight activities (i.e. number of movements for each mosquitoes) was modeled with mixed model using a negative binomial, the sex of mosquitoes, and infection status toward *A. taiwanensis* as fixed factors and the experimental replicates as random variables. The impact of the fixed factors was tested with a type II Wald χ^2^ test. The datasets and scripts used for statistical analysis have been deposited on zenodo (https://zenodo.org/record/8252320).

## Results

### Recently introduced mosquitoes in mainland France harbor lower prevalence of *A. taiwanensis*

**Fig. 1.**
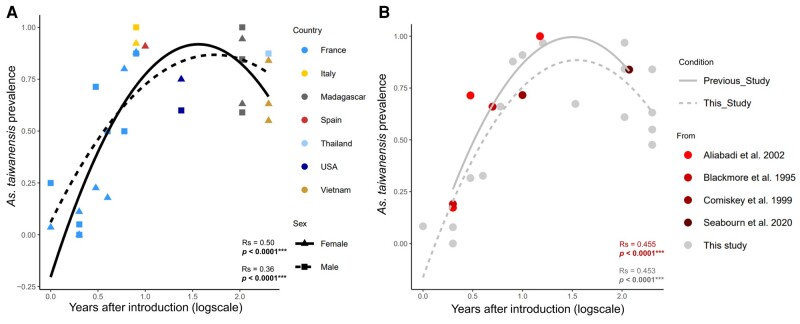
Correlation between the prevalence of *A. taiwanensis* and the time after introduction of the mosquito populations in each sampling site. A) Correlation between parasite prevalence (i.e. proportion of infected individuals) and the population ages (number of years separating the mosquito introduction from the collection date) for females (triangle) and males (square) has been investigated in different countries. B) A comparative analysis has been realized between previously published data and the data from this study regarding the correlation between parasite prevalence and population ages. The data from previously published studies include larvae (circles) and mixed adults (diamond). Each dot represents a sampling site. Native populations were arbitrarily set at 200 years to be ranked at the end of the *x*-axis.

The prevalence of *A. taiwanensis* was estimated in 598 mosquitoes sampled among 17 populations using diagnostic PCR (Table S1, Fig. S1). Samples collected in mainland France were a combination of recently introduced populations and intermediate ones. For all other countries, native or intermediate populations were collected. Among the studied variables, the population origin significantly impacted the prevalence of the parasite and differences were observed between sexes along the sampling sites (Table 1). For those reasons, the two sexes were then analyzed separately. To explain features of the mosquito origin that can influence the prevalence of the parasite, we further tested the impact of climate, population age, and geographical distance between mosquito populations. Variations in climatic regions have no significant impact on parasite prevalence (Table 1, Fig. S2). However, it was positively correlated with the mosquito population age for both males and females (Table 1, Fig. 1A and B). In recent mosquito populations (<2 years), the observed prevalence of *A. taiwanensis* ranged from 0 to 25%, while it ranged from 37 to 100% in populations that were native or introduced for more than 6 years. This effect was specifically strong on French sites that are geographically closed from each other but harbor high differences in parasite abundance correlating with differences in population ages. We could therefore assume that spatial autocorrelation might influence this observed pattern. The Moran's index revealed a very weak but significant spatial autocorrelation for mosquito populations at the global scale for both female and male mosquitoes (*I* = 0.27, *P* = 0.038; *I* = 0.28, *P* = 0.013 for both sexes, respectively). When considering the French populations separately, no significant autocorrelation was evidenced for females (*I* = 0.05, *P* = 0.064) and a very weak and yet significant autocorrelation was evidenced for males (*I* = 0.16, *P* = 0.02). To separate the impact of population age from that of geographical distances, prevalence data from previously published studies concerning recently introduced and older populations from the United States were considered (Fig. S1, Table S2). The extracted data referred to prevalence of *A. taiwanensis* in mosquito larvae and mixed adults. No spatial autocorrelation was observed when analyzing this new dataset exclusively (*I* = −0.52, *P* = 0.22), while the parasite prevalence and population age were positively correlated (Fig. 1B). When merging both datasets, prevalence of the parasite was still correlated with the age of the mosquito population (*R*s = 0.46, *P* < 0.001) in absence of spatial autocorrelation (*I* = 0.11, *P* = 0.34).

**Table 1.** Summary of statistical analyses.

Category	Variable	Factor	Test	Details on sample	Subsample	χ²	*P-*value	Index
Field	Prevalence	Origin	GLM			280.65	**<0**.**0001**^a^	
		Sex				0.46	0.496	
		Origin × Sex				25.04	**0**.**008**^b^	
		Origin			Female	198.09	**<0**.**0001**^a^	
					Male	107.61	**<0**.**0001**^a^	
		Climate				0	1	
		Climate × Sex				0	1	
		Age of the population	Spearman		Female		**<0**.**0001**^a^	0.50
					Male		**<0**.**0001**^a^	0.36
		Geographical distance	Moran		Female		**0**.**038**^c^	0.27
					Male		**0**.**013**^c^	0.28
					France × Female		0.064	0.05
					France × Male		**0**.**020**^c^	0.16
					USA^d^ × Mixed		0.688	−0.38
Experiment		Egg desiccation	GLMM*^b^*	Desiccation resistance	Female	20.124	**<0**.**0001**^a^	
					Male	17.456	**<0**.**0001**^a^	
		Adult mosquito density		Interindividual transmission	Female	NA	NA	
					Male	NA	NA	
		Larval mosquito density		Density-dependant prophylaxis	Female	NA	NA	
					Male	NA	NA	
Field	Relative abundance	Years after introduction	Spearman	Ascogregarina|Otu000001	Female		**0**.**034**^c^	0.18
					Male		0.465	−0.07
				Ascogregarina|Otu000002	Female		**<0**.**0001**^a^	0.51
					Male		**<0**.**0001**^a^	0.50
				Ascogregarina|Otu000003	Female		**0**.**0008**^a^	0.28
					Male		**<0**.**0001**^a^	0.40
		Geographical distance	Moran	Ascogregarina|Otu000001	Female		0.371	0.09
					Male		0.246	0.14
					France × Female		**0**.**034**^c^	−0.43
					France × Male		0.107	0.00
				Ascogregarina|Otu000002	Female		**0**.**005**^b^	0.48
					Male		0.316	0.03
					France × Female		0.576	−0.31
					France × Male		0.158	−0.03
				Ascogregarina|Otu000003	Female		**0**.**013**^c^	0.48
					Male		0.426	0.03
					France × Female		0.127	−0.39
					France × Male		0.956	−0.24
	Absolute abundance	Years after introduction	Spearman	Ascogregarina|Otu000001	Female		0.87	−0.04
					Male		0.51	0.15
				Ascogregarina|Otu000002	Female		**0**.**007**^b^	0.60
					Male		**0**.**007**^b^	0.55
				Ascogregarina|Otu000003	Female		**0**.**001**^a^	0.63
					Male		**0**.**005**^b^	0.62
Experiment	Parasite intensity	Egg desiccation	GLMM^e^	Desiccation resistance	Female	0.75	0.387	
					Male	9.91	**0**.**002**^b^	
	Parasite abundance	Adult mosquito density		Interindividual transmission	Female	15.07	**0**.**001**^a^	
					Male	20.535	**<0**.**0001**^a^	
		Larval mosquito density		Density-dependant prophylaxis	Female	3.10	0.0784	
					Male	1.14	0.2855	
	Flight activity	Infection status	GLM	Locomotor activity system	Female	11.58	**0**.**0151**^c^	
					Male	0.48	0.1988	

^a^ *P* ≤ 0.001. ^b^*P* ≤ 0.01. ^c^*P* ≤ 0.05. ^d^Experimental replicates were used as random variable. ^e^Data extracted from the literature. Statistically significant *P*-values are in bold.

### *Ascogregarina taiwanensis* abundances correlate with the microbiota composition, the mosquito origin and the population age

**Fig. 2.**
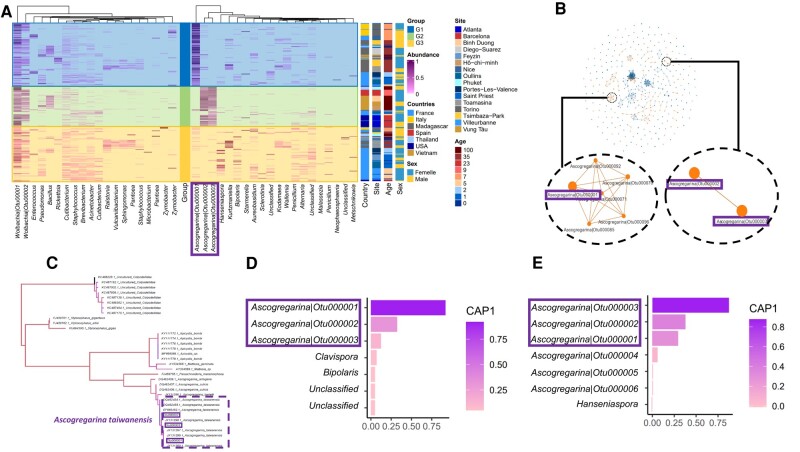
Heatmap of the microbiota associated with *A. albopictus*. A) The 20 most abundant prokaryotic and eukaryotic OTUs were represented in columns. The number attached to *Wolbachia* and *Ascogregarina* OTUs corresponds to the OTUs number in the following analysis. The “Abundance” scale shows the OTU proportion in the community. Rows are classified depending on the central scale that represent three different groups based on eukaryotic community dissimilarity (Bray–Curtis distance) and that correlates with variations in the three *A. taiwanensis* OTUs proportions. Individuals belonging to the G1 group are dominated by *Ascogregarina* Otu000001, those belonging to the G2 group are dominated by *Ascogregarina* Otu000002 and *Ascogregarina* Otu000003, and those belonging to the G3 group are not dominated by *Ascogregarina* OTUs. Three additional scales correspond to the individuals' origin site, age (years after introduction), and sex. B) A network analysis showing positive correlations between microbial OTUs (eukaryotes are represented in light while prokaryotes are in dark and each link represents a correlation with *ρ* > 0.25). The left network in the zoom is dominated by the *A. taiwanensis* Otu000001 and is correlated with minor OTUs assigned to *Ascogregarina*. The right network in the zoom shows correlations between the *A. taiwanensis* Otu000002 and Otu000003. C) Phylogenetic analysis revealed the belonging of OTU identified as Gregarinasina to the species *A. taiwanensis*. D) The main eukaryotic OTUs according to the coordinate of the axis (CAP1) extracted from a constrained analysis of principal coordinates (Capscale) on the OTU relative abundance highlighting correlations between OTUs and the age of the population. The three *A. taiwanensis* main OTUs are circled. E) The main eukaryotic OTUs according to the coordinate of the axis (CAP1) extracted from a constrained analysis of principal coordinates (Capscale) on the OTU absolute abundance highlighting correlations between OTUs and the age of the population. The three *A. taiwanensis* main OTUs are circled in violet.

To compare dynamics of *A. taiwanensis* within the microbiota of the different mosquito populations, a metabarcoding analysis was conducted on a subset of 248 male and female mosquitoes (Table S1). Mosquitoes were randomly selected for this analysis within the previously described populations. The eukaryotic and prokaryotic microbiota of *A. albopictus* were, respectively, dominated by two OTUs classified as *Wolbachia* (Fig. 2A) and three OTUs classified as *Ascogregarina* (Fig. 2A). In total, 8 different OTUs of *Ascogregarina* were identified among the 200 most frequently retrieved OTUs but 5 showed low abundances. Those three dominant *Ascogregarina* OTUs showed a prevalence of 74.4% and an average abundance of 42.1 ± 41.2% (mean ± SD) among the eukaryotic microbiota, while the two dominant *Wolbachia* OTUs showed a prevalence of 93.4% and an average abundance of 39.4 ± 34.4% among the prokaryotic microbiota. Other OTUs identified as *Aureobasidium* and *Penicillium* genera for eukaryotes or as *Cutibacterium*, *Staphylococcus*, and *Acinetobacter* genera for prokaryotes were also prevalent. Study of factors involved in variation of microbiota composition (i.e. β-diversity) between individuals revealed that the origin of mosquitoes was the most important (*R*^2^ = 0.32, *P* = 0.001) structuring factor in interaction with the sex (*R*^2^ = 0.04, *P* = 0.001) at a lower magnitude (see Table S3). Both eukaryotic and prokaryotic microbiota dissimilarities were also correlated with the age of the population (Fig. S2), climate (Fig. S3), and geographical distance between individuals (Fig. S4).

**Fig. 3.**
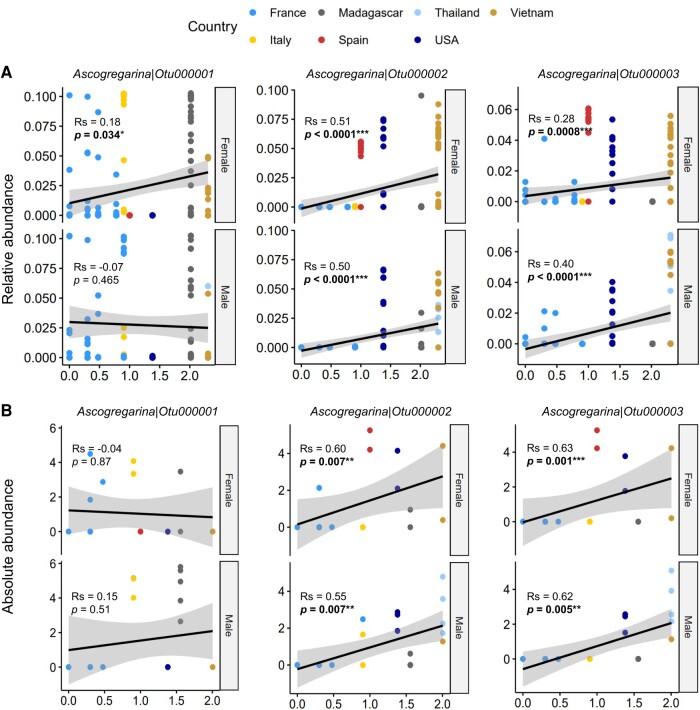
Correlations between *Ascogregarina* OTUs abundance and the mosquito population age. A) The age of the mosquito populations and the relative abundance of the three dominant *Ascogregarina* are represented for female and male mosquitoes. B) The age of the mosquito populations and the absolute abundance of the three dominant *Ascogregarina* are represented for female and male mosquitoes. The *P*-value represents the significance of the spearman correlation between OTUs absolute abundances and the age of the population (number of years between introduction and sampling). The native population was set to 200 years after introduction to be ranked at the end of the *x*-axis.

Based on their similarity, the eukaryotic community could be separated in three groups (Fig. 2A). The first group (60 individuals) was dominated by the most prevalent OTUs of Ascogregarina (Otu000001), the second (43 individuals) by two different OTUs of Ascogregarina (Otu000002 and Otu000003), and the last one (84 individuals) by neither of those 3 OTUs. This pattern was confirmed with correlations networks that showed two independent networks of positive correlations between relative abundances of OTUs identified as Ascogregarina (Fig. 2B). The first network involved the dominant Ascogregarina (Otu000001) and fifth other underrepresented Ascogregarina OTUs (Otu000070, Otu000071, Otu000085, Otu000092, Otu000096), while the second one involved solely the two others dominant Ascogregarina OTUs (Otu000002 and Otu000003). The three dominant Ascogregarina OTUs could be confidently classified within the *A. taiwanensis* species based on a maximum likelihood phylogeny conducted on this same 18S rDNA region (Fig. 2C). The three most abundant *A. taiwanensis* OTUs were explaining most part of the eukaryotic microbiota that covaries with the age of the population based on a CAP analysis (Fig. 2D). Correlations of relative abundances of those three OTUs showed that Otu000002 and Otu000003 increased in abundance in old populations, while Otu000001 significant vary with the age of the population only for females with a low correlation index (Fig. 3A). For each of the three group of microbiota similarity, absolute abundances were calculated for a subset of 7 females and 7 males. When converted in absolute abundances, the three most abundant *A. taiwanensis* OTUs were still the most discriminant ones covarying with the age of the population in the CAP axis (Fig. 2E). Otu000002 and Otu000003 showed a significant positive correlation between their absolute abundance and the age of the mosquitoes’ population, while this was not significant for Otu000001 (Fig. 3B).

### Parasitic success decreases over time when desiccated eggs are stored during human-aided transportation

**Fig. 4.**
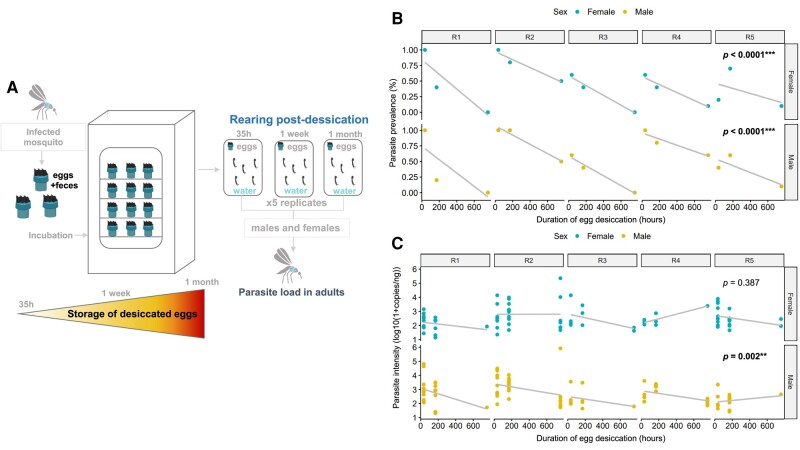
Impact of egg maintenance outside of water (desiccated) on the infectivity of *A. taiwanensis*. A) Desiccated eggs have been stored for 35 h, 1 week, or 1 month in a container before being flooded to trigger egg hatching. Five containers were tested for each time point. The parasite abundance was estimated on emerging adults by qPCR. B) The impact of desiccation time on parasite prevalence was represented for each repeat (R1 to R5) and both sexes. C) The impact of desiccation has also been tested on the parasite intensity and was represented for each repeat (R1 to R5) and both sexes. The *P*-value obtained from a Wald χ^2^ test conducted on a GLMM model represents the significance of the slope.

To evaluate whether egg desiccation may restrain the parasite infectivity through long-distance transportation, we collected eggs from five containers. Desiccated eggs were separated into three groups (100 eggs/group) that we, respectively, stored 35 h, 1 week, and 1 month before being flooded (Fig. 4A). Parasite quantifications in adults were performed on 10 individuals per time point and replicate. Due to the high number of uninfected mosquitoes after desiccation, parasite prevalence and intensity were analyzed separately. Parasite prevalence decreased when desiccation duration increased for both males and females (Fig. 4B, Table 1). This treatment also affected the parasite intensity within infected males (Fig. 4C, Table 1).

### Founder effects can reduce the amplitude of parasite infections due to low mosquito population densities and an absence of DDP

**Fig. 5.**
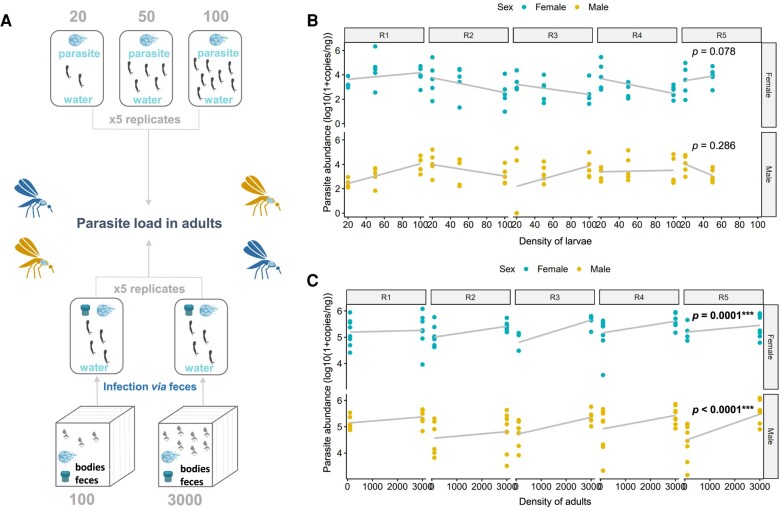
Density of adults releasing oocysts positively impacts the gregarine colonization and it is not counter balanced by DDP. A) The impact of adult and larval mosquito density on the parasite success has been tested through two different experiments. In a first experiment, the density of adults releasing oocysts in water habitats was controlled. They were allowed to release oocysts through feces and body of dead individuals for a week before rearing larvae. In a second experiment, we tested DDP. To that end, larvae were infected with the same water for 2 days before being separated in batches of 20, 50, and 100 individuals in 100 mL of water. Each experiment was repeated 5 times. The parasite abundance was estimated on emerging adults by qPCR. B) The impact of adults density that released oocysts in the larval water habitat and C) the impact of larval density (i.e. number of larvae per 100 mL of water) on the parasite abundance was represented for each repeat (R1 to R5) and both sexes. Each dot represents a single mosquito. The *P*-value obtained from a Wald χ^2^ test conducted on a GLMM model represents the significance of the slope.

Two experiments were designed to determine whether the density of adults releasing oocysts and DDP (i.e. number of larvae receiving oocysts) could influence the parasite success to colonize new adult mosquitoes (Fig. 5A). When the number of adults releasing oocysts in water was increased, we observed that all the individuals were infected but higher parasite intensity could be reported (emmeans estimate ± SE: 207 ± 45.7 and 191 ± 49.8 for males and females, respectively) in individuals emerging from the water habitat (Fig. 5B, Table 1). We also noticed that emerging females harbored more parasite oocysts than males (emmeans estimate ± SE: 100 ± 33.5). However, the parasite success was not significantly modified by larval densities since all the individuals were infected and variations in parasite intensities could not be consistently explained by the concentration larvae in the container (Fig. 5C, Table 1).

### The parasite is not likely to impair mosquito dispersion through flight since parasitized individuals are more active

**Fig. 6.**
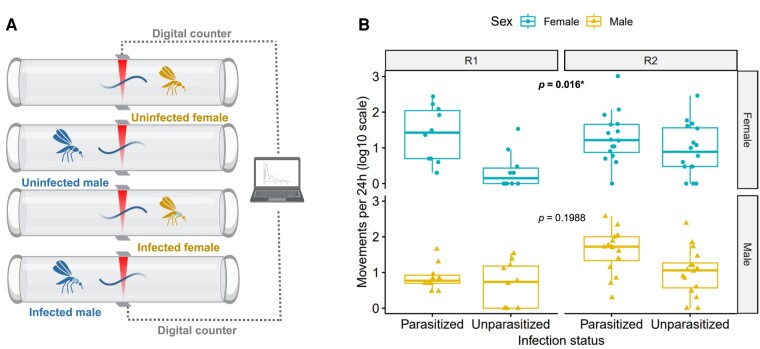
*Ascogregarina taiwanensis* infection impacts on mosquito flight activity. A) Parasitized or unparasitized mosquitoes activity was measured in a locomotor activity monitor. Movements were recorded for 24 h by measuring the number of times each individual pass through an infrared beam. The experiment was repeated on two generations. B) The impact of parasitism on flight activity (i.e. number of time they passed in front of an infrared beam during 24 h) was measured for two generations (R1 and R2) and both sexes. The *P*-value obtained from a Wald χ^2^ test conducted on a GLMM model represents the significance of the slope.

The flight behavior of uninfected and infected mosquitoes was assessed using a locomotor activity monitor. We recorded individually the number of movements of parasitized and unparasitized mosquitoes during 24 h (Fig. 6A). Contrarily to our expectations, parasitized mosquitoes were significantly more active than unparasitized ones (emmeans estimates: 1.34 ± 0.45 and 0.89 ± 0.45 for females and males, respectively; Fig. 6B, Table 1). Although it was not statistically significant, the same pattern was observed for parasitized males (49.4 moves) in comparison to unparasitized ones (18.9 moves). Therefore, those results suggest that the low infection rate of mosquitoes in young populations is not related with a higher propensity of uninfected individuals to disperse by flight but the parasite could, conversely, promote the mosquito flight activity.

## Discussion

In this study, we reported a positive correlation between *A. taiwanensis* prevalence and *A. albopictus* population age for different mosquito populations. Such parasitism escape has been evidenced in particular within recently introduced populations in France and United States. The same pattern was observed regarding the abundance of 2 out of the 3 main *A. taiwanensis* genotypes identified in the metabarcoding analysis. All in all, those convergent tendencies suggest that *A. albopictus* might have escaped its natural parasite multiple time along its invasion history. Such decrease might result from subsampling of noninfected or poorly infected individuals in the source population that formed the introduced ones. However, this is more likely to occur if the parasite is rare in the source population (16, 17), while our field study suggests that the native or old populations sampled along potential source areas are highly colonized by the parasite. Indeed, most recent mosquito records and population genetic studies suggest that mosquito introduction in mainland France is the combination of south European, north-east American introduced populations as well as old invasive populations from the Indian Ocean and native populations from Asia (74, 75). Based on PCR diagnostics and microbial community analysis on populations from those areas, we can argue that the parasite is likely to be prevalent and dominant in source population. Three dominant genotypes of *A. taiwanensis* were observed in those area and two of them (Otu0002 and Otu0003) correlated with the laps time separating the mosquito introduction to its sampling. Indeed, metabarcoding and qPCR analysis suggest that those two OTUs dominate the microbiota of intermediate and native populations but not that of recently introduced ones. A recent study on parasite infection in invasive species has shown that parasite characteristics are reliable to predict parasite retention (76). The authors even demonstrated that specialist parasites (like *A. taiwanensis*) are usually more persistent to coinvade with their native host. All in all, we cannot exclude that stochasticity due to subsampling of noninfected individuals may have played a role in the absence of infection in recently introduced populations but since it is unlikely, we argue that other mechanisms have probably led them to escape *A. taiwanensis.*

The Asian tiger mosquito invasive process is mainly induced by human-mediated large-scale dispersal of desiccated eggs as previously discussed (77) and is therefore facilitated by desiccated egg ability to resist without hatching during transportation (24, 78). The desiccation and storage laps time that we selected (i.e. up to 1 month) were included within the range the eggs can survive without showing consistent decrease in survival (40). We demonstrated that the abundance and prevalence of parasite infection in mosquitoes hatched from desiccated eggs decrease as a function of the time the eggs were kept before being flooded. Similarly, the gregarine *Apolocystis elongata* showed a reduced infectivity toward its earthworm host *Eisenia foetida* 3 weeks after oocysts have been released on a dry soil (79). Sensitivity to desiccation has also been reported as a major oocyst weakness in related Apicomplexan species such as *Cryptosporidium* or *Eimeria* species (80, 81). The molecular mechanism involved in desiccation sensitivity has poorly been studied until now in Apicomplexa but is likely to be due to the peculiar evolution of those microorganisms that recently diverged from marine algae (82). At the opposite, resistance of cysts to desiccation has been acquired in some Apicomplexan taxa such as *Toxoplasma* spp. and was associated to the production of late embryogenesis abundant-related proteins (83). The particular biochemical properties of those protein enable them to harbor chaperone-like activities and contribute to protein stabilization that may reinforce the cyst wall integrity and to limit water loss. The lack of acquisition of such protein in *A. taiwanensis* still needs to be demonstrated but may explain why this parasite that is often released in water container poorly resist to desiccation.

Since transportation of desiccated eggs is a major path driving the mosquito introduction (28–32, 74, 84), human-aided transportation could be a consistent factor explaining *A. albopictus* parasitism escape after mosquito population introduction. However, part of the mosquitoes are also transported as adults for a shorter period of time in car cabin and this path that can hardly be related to parasitism escape (29). Habitat and host permissiveness to the parasite after introduction may also be a key factor that could have decreased the infection probability for a short period of time after mosquito introduction (85). Those processes are complex and may involve biotic and abiotic components. We did not fully address this question and argue that further studies must be conducted to better understand the importance of genotype × genotype × environment (G × G × E) interactions in *A. albopictus—A. taiwanensis* interactions. However, our results provide new insights for further investigations. Firstly, the metabarcoding of eukaryotic and prokaryotic communities in *A. albopictus* did not evidence that the parasite was replaced by another competitive parasite from the invasion range. This part suggests that the biotic part associated to microorganism does not seem very relevant to explain the host–parasite dynamics. Secondly, the habitats in mainland France and in United States could be partially refractory to the parasite since we evidenced for both localizations’ correlation between geographical distances and parasite prevalence. Further studies on biotic and abiotics are necessary to conclude on the environment importance on this host–parasite relationship.

In other host–parasite systems such as snail–trematode, the host density has been shown to be a good predictor of parasite colonization success (86). Such phenomena are often predicted to occur for horizontally transmitted parasites due to interindividual contacts leading to higher transmission probability that increase with population density (87). Since interindividual horizontal transmission of parasites is mediated through oocysts released from adults in water habitats, one can expect that a reduced number of mosquitoes invading a new area would result in a limited number of parasites in the breeding sites to colonize new hosts. Previous studies on *A. taiwanensis* evidenced that the parasite oocyst load released in water influence the success of host infection (59). In this study, we have complemented those results by showing that the number of adult mosquitoes releasing parasites positively increases the number of parasites efficiently colonizing unparasitized conspecifics emerging from water habitats. Interestingly, an interseasonal mosquito and *A. taiwanensis* dynamics have been shown to be correlated over the year (51). Those results converge toward the importance of high mosquito population densities for an efficient parasite transmission, independently from the probability of parasite acquisition. Previous studies on gregarious insects showed that an adaptive response to host high density leads to a reduction of the parasite susceptibility through the enhancement of their immune system (22). This phenomenon is also named DDP. For instance, in response to density raise, the bumble bee *Bombus terrestris* (Hymenoptera) and the velvet bean caterpillar *Anticarsia gemmatalis*, increase their phenoloxidase activity (immune response leading to a melanization process) (88, 89). In this study, we have shown an absence of larval density-mediated modulation of the parasite infection cycle. Although the immune response has not been tested, we can then predict that the parasite transmission drastically increases with the population size without being slowed down by DDP.

We have highlighted the impact of the different invasion processes in the host–parasite relationship. Contrarily to our hypothesis, local mosquito dispersal through flight has been shown to be improved by parasitism. Local flight distance of field populations is generally limited to a few hundred meters (47) and remains a secondary factor to explain *A. albopictus* invasiveness. The hypothesis of gregarine dispersal through local flight is debatable due to opposite examples. Indeed, the infectivity of the gregarine *O. elektroscirrha* to monarch butterfly (*D. plexippus*) has been proved to reduce host wing mass and resistance (90). In the dragonfly (*Libellula pulchella*), gregarines are also negatively affecting the flight performance by decreasing the concentration of muscle myosin and impairing muscle contractile performance (91, 92). The results reported here are preliminary. Indeed, laboratory experiments on mosquitoes behavior using locomotor activity monitor do not take into account various stimuli (e.g. breeding sites, hosts, etc.) from the environment that might differentially enhance the local dispersal of mosquitoes (93, 94). Other factors such as the mosquito age and nutrition would also be considered in the future, while previous studies showed that the activity varies according to those factors (95). If our result is confirmed under more complex conditions, this would suggest that mosquitoes that have been colonized by the parasite after introduction might be more prompt to locally disperse.

Recently, a mathematical model predicted *A. albopictus* competitiveness against the tree hole mosquito *Aedes triseriatus* in presence of gregarine parasites (96). In most scenarios, *A. albopictus* dominates *A. triseriatus*, but *A. taiwanensis* infection may decrease the competitiveness of its host by increasing the larval development time and inducing a higher mortality, according to other studies (66). The authors concluded that parasitism reduces *A. albopictus* competitiveness. Selective pressure induced by competition might explain the loss of parasites in recently introduced *A. albopictus* populations since its presence might restrain the population settlement probability. Another hypothesis to explain the parasitism escape is a dilution effect when *A. albopictus* colonizes new areas. Both parasite prevalence and intensity could be reduced due to a wider range of potential host (induced by native mosquito species). Indeed, they can colonize first instar larvae of other mosquito species but rarely achieve to complete their life cycle and replicate inside them (49). To the best of our knowledge, no study evidenced dilution of *A. taiwanensis* out of *A. albopictus*. However, dilution effect of *Ascogregarina barreti* has been experimentally observed during co-inoculation of its native host *A. triseriatus* with other mosquito species such as *Aedes japonicus* (97) and *A. albopictus* (98). It was also suggested by field surveys reporting that the prevalence of *A. taiwanensis* colonizing *A. albopictus* was higher when the mosquitoes did not share breeding sites with *A. aegypti* (99)*. A albopictus* is often reported as a strong competitor toward other mosquitoes (100–105). Therefore, the parasite prevalence dynamics in young populations could be due to an evolution of the dilution effect resulting from a higher diversity of mosquitoes in breeding sites at the early stage of the colonization process that decrease over the time due to interspecific competition. Field studies on the arthropod's community sharing breeding sites with recent and ancient populations may help to find relevant alternative hosts to test this hypothesis.

## Conclusion

In this study, we provide new insights into the dynamics of *A. Albopictus–A. taiwanensis* relationship by combining field observations and laboratory experiments. In French populations, the mosquito host has escaped its parasite after introduction and this process is unlikely to be stochastic. The maintenance of nonflooded eggs in containers during human-aided transportation and the decrease in mosquito founding population after introduction have likely altered the parasite maintenance and transmission. Local dispersal through flight is probably not affected by the parasite since the flight activity of unparasitized individuals was lower than that of their parasitized conspecifics. We need further investigations on the consequences of the rupture of this natural interaction to understand how it may affect the mosquito ecology, population dynamics, and pathogen transmission.

## Supplementary Material

pgae175_Supplementary_Data

## Data Availability

All the reads, datasets, and scripts used for this analysis have been deposited on Zenodo (https://zenodo.org/record/8252320).

## Preprints

This manuscript was posted on a preprint: https://doi.org/10.1101/2023.02.20.529246.
