# Supplementary material for: Human-aided dispersal and population bottlenecks facilitate parasitism escape in the most invasive mosquito species
Source: PNAS Nexus. 2024 Apr 30;3(5):pgae175. doi: 10.1093/pnasnexus/pgae175 (PMC11074241; doi:10.1093/pnasnexus/pgae175)
Supplement: pgae175_Supplementary_Data [file pgae175_supplementary_data.docx]

**Supplementary material.**

***DNA extraction***

Mosquito samples were rinsed three time with sterile water, surface disinfected 5min in ethanol and washed 5 times in sterile water. DNA was extracted from each mosquito individually crushed with three 3mm borosilicate beads and under agitation during 60s at 6m/s with a Fastprep homogenizer (MP Biomedicals). Homogenate was then suspended into a CTAB extraction buffer (2% hexadecyltrimethyl ammonium bromide, 1.4 M NaCl, 0.02 M EDTA, 0.1 M Tris pH 8.0, 0.2% 2-β-mercaptoethanol) and heated for 2h at 60°C. After incubation, 2mg/ml of RNase was added to the extraction buffer and incubated at 37°C for 5 min. DNA was separated from proteins and lipids with an equivalent volume of phenol-chloroform-isoamyl alcohol (25:24:1, v:v:v), vortexed for 15min and centrifuged for 30min at 16,100g. Aqueous phase was harvested and mixed with an equivalent volume of chloroform:isoamyl alcohol (24:1, v:v). The mixture was vortexed for 15min and centrifuged for 30min. The newly separated aqueous phase containing the nucleic acids was harvested. DNA was precipitated with isopropyl alcohol and centrifuged for 30min at 16,100g. DNA pellet were washed with 70% cold ethanol and centrifuged for 30min at 16,100g. Ethanol was removed and DNA was suspended in 20µl of sterile water. DNA extracts were quantified with a spectrophotometer (SAFAS) and diluted at 15ng/µl.

***qPCR estimation of the eukaryotic and prokaryotic microbial density***

Since variations in OTU abundances may be influenced by variations in absolute densities of microorganisms, qPCR essays were performed on samples for which variations in *As. taiwanensis* abundances were observed. Following the metabarcoding analysis and correlation network analysis, three different groups of *Ae. albopictus* microbiota were observed depending on their infection status with *As. taiwanensis* (see Results section). Prokaryotic and Eukaryotic qPCR essays were performed on 7 males and 7 females from each of those three groups using the same primers than in metabarcoding. Reaction mix contained 1X of SYBR Green iTaq (Bio-Rad), 3µM of each primer and 5ng of DNA. PCR cycles were performed on a CFX96 Touch Real-Time PCR detection system (Bio-Rad) following a 3min initial denaturation at 95 °C followed by 40cycles with 5s of denaturation at 95°C and 1min of hybridization and amplification at 60°C. A final denaturation of the PCR products was conducted by progressively increasing the temperature from 60°C to 90°C at 0.11°C/s. The Ct values were used to quantify the copies number of each gene with a standard range of 10^7^ to 10^0^ genomic DNA copies of *Escherichia coli* DH5-α for the 16S rDNA and a 10^7^ to 10^0^ genomic DNA copies of *Saccharomyces cerevisiae* CEN.PK2-2 for the 18SrDNA.

***PCR-based estimation of* Ascogregarina taiwanensis *prevalence***

Detection of *As. taiwanensis* in DNA from field collected individuals was conducted with diagnostic PCR using primers targeting the ITS1-5.8SrDNA-ITS2 intergenic region with the previously published AU (5’-ACCGCCCGTCCGTTCAATCG-3’) and AT (5’-GAGAAGCCGTCGTCAATACAGC-3’) primers (Morales et al., 2005). The mix contained 1X of Hoststart Master mix (Dutscher, 0.2ng/ml of BSA (Promega), 0.1µM of each primer, 0.1X of GC-Enhancer (Biofidal) and 30ng of mosquito genomic DNA. Reactions were performed with a 10min initial denaturation at 96°C, followed by 35 cycles of amplification including 20s at 96°C, 1min at 50°C, 2min at 72°C and a final elongation of 10min at 72°C. Amplification signals were identified under UV light on a Geldoc 2000 system (Biorad) after 20min of electrophoresis migration at 100V on a 1.5% agarose gel colored with Ethidium Bromide.

***Design of qPCR primers for the quantification of* Ascogregarina taiwanensis**

Primers hybridizing *As. taiwanensis* were designed *in* *silico* based on an alignment of previously published 18S rDNA sequences of Apicomplexa (genebank accession numbers JX131299.1, DQ462455.1, DQ462454.1, JX131298.1, JX131300.1, JX131297.1, EF666482.1, JX131296.1, DQ462456.1, DQ462457.1, DQ462458.1, DQ462459.1, LC109003.1, AY179988.1, AY789075.1, AF129883.1, HQ224956.1, AB071177.1, DQ176427.1, EF024130.1, EF024388.1, EF024744.1, EF024808.1, EF024116.1, EF024963.1, EF024809.1, EF024338.1, EF024673.1, EF024471.1, EF024351.1, EF024347.1, EF024539.1, AY334569.1, KF130567.1, AY334568.1, AY490099.1, AY919720.1, AY919821.1, AY919809.1, KF130566.1, KF130601.1, EU910605.1, EF024102.1, AJ243064.2, KT072989.1, MF998086.1, FN546182.1, FJ459739.1) to select a conserved region in *As. taiwanensis* that diverged from other species. Two specific primers named AscoJSF (5’-CTMGGCTTGACTTCGGTCT-3’) and AscoJSR (5’-TTCCATGCTGGAGTATTCAAGG-3’) were designed and produced a fragment of 150bp. A standard concentration of the fragment cloned in a vector was prepared. It was first amplified with a PCR mix containing 1X of buffer, 1.5mM of MgCl_2_, 200µM of dNTP, 200nM of each primer, 0.625 Units of Taq DNA Polymerase (New England Biolabs) and 30ng of DNA from a parasitized mosquito individual. The PCR program was ran with 95°C of initial denaturation for 4min, followed by 35 cycles including 30s at 95°C, 30s at 56°C, 15s at 68°C and a final elongation of 5min at 68°C. The PCR product was purified with a PCR purification kit MinElute (Qiagen), cloned into a PCR 2.1-TOPO TA vector (Invitrogen) and transferred into competent *Escherichia coli* Top10 cells (Invitrogen) following manufacturers’ recommendations. Competent cells containing the vector and the insert were selected on LB agar supplemented with 25µg/ml of Kanamycin and 20µg/ml of Xgal and 23.8µg/ml of IPTG after 24h of incubation at 37°C. Presence of the insert was also verified with PCR on white clones that developed on the medium. DNA from the competent cells was extracted with a DNeasy Blood and Tissue kit (Qiagen) following manufacturers’ recommendations and linearized 1h at 37°C in a mix containing 0.2µg/µl of DNA, 1X of SurE/Cut Buffer B and 10U of BamH1 endonuclease (Roche). Reaction was inactivated at 65°C for 15min. This digested DNA was purified with a MinElute PCR purification kit (Qiagen), quantified with Quanti-it dsDNA BR Assay kit (Invitrogen) and used as a standard for qPCR with concentrations ranging from 10^8^ to 10^0^ copies. Reaction mixes for quantification included 0.5µM of each primer, 1X of iTaq Universal SYBR Green Supermix (Bio-Rad) and 5ng of DNA. The qPCR program included a 5min initial denaturation at 95°C, followed by 40 cycles with 15s at 95°C, 30s at 56°C and 10s at 60°C followed by a progressive denaturation of the qPCR products from 65°C to 97°C at 0.11°C/s.

***Metabarcoding of eukaryotic and prokaryotic microbial communities***

Both PCR amplifications were conducted under sterile conditions with 1X of Hotstart 5X Bioamp Master Mix (Biofidal), 0.2µM of each primers, 0.2mg/ml of Bovine Serum Albumin (New England Biolabs), 0.4X of GC rich Enhancer (Biofidal). For 16S rDNA, PCR program started with 10min of initial denaturation at 96°C followed by 35 cycles including 20s of denaturation at 96°C, 1min of annealing at 54°C, 30s of extension at 72°C and ended with a final extension of 10min at 72°C. For 18S rDNA, PCR program started with 10min of initial denaturation at 96°C followed by 35 cycles including 20s of denaturation at 96°C, 30s of annealing at 54°C, 30s of extension at 72°C and ended with a final extension of 10min at 72°C. Three negative controls corresponding to blank extractions and PCR were included for each gene.

Details concerning the mother pipelines used for bioinformatics treatment: A total of 12,836,921 and 14,248,382 reads were obtained from 16S and 18S rDNA respectively and were demultiplexed using the Mothur pipeline (69). Reads were trimmed based on size (270-310bp and 400-550bp for the 16S and 18S rDNA, respectively) and sequences were conserved if they contained no ambiguous position and if both strands of the paired-end reads aligned together. Chimeras were removed using CHIMERAVSEARCH. The alignment was performed using Silva v.132 database for bacteria and eukarya and reads that did not align were filtered out. However, for sequences that matched gregarine, a supplemental local alignment (BLAST) was performed against the Genbank database due to the lack of reference sequences in Silva. Clustering was performed using Opticlust method allowing a maximum dissimilarity rate of 3%.

**Table S1. Aedes albopictus population used in the current study.**

| **Country** | **Origin site** | **Months of sample** | **Years of sample** | **Years after introduction** | **Sex** | **n. samples prevalence** | **n. samples metabarcoding** | **Climate** | **GPS coordinates** |
| --- | --- | --- | --- | --- | --- | --- | --- | --- | --- |
| France | Saint-Priest | July | 2012 | 0 | males | 8 | 10 | Semi-continental | N : 45°41'49'' |
|  |  |  |  |  | females | 28 | 10 |  | E : 4°58'50' |
|  | Saint-Priest | August | 2018 | 1 | males | 24 | - | Semi-continental | N: 45°43'8' |
|  |  |  |  |  | females | 28 | - |  | E : 4°55'9'' |
|  | Oullins | July | 2018 | 1 | males | 20 | 10 | Semi-continental | N: 45°42'42'' |
|  |  |  |  |  | females | 18 | 10 |  | E : 4°48'2'' |
|  | Feyzin | July | 2018 | 1 | males | 18 | 10 | Semi-continental | N: 45°40'25' |
|  |  |  |  |  | females | 22 | 10 |  | E : 4°51'33'' |
|  | Villeurbanne | July | 2018 | 5 | males | 26 | 10 | Semi-continental | N : 45°46'18'' |
|  |  |  |  |  |  | 30 | - |  | E : 4°53'24'' |
|  | Nice | August | 2012 | 7 | males | 16 | - | Mediteraneen | N : 43°41'60'' |
|  |  |  |  |  | females | 20 | 10 |  | E : 7°17'33'' |
|  | Portes-Les-Valence | July | 2013 | 2 | males | 7 | 10 | Semi-continental Mediteraneen | N : 44°52'8'' |
|  |  |  |  |  | females | 31 | 10 |  | E : 4°52'9' |
| Italy | Turin | September | 2011 | 15 | males | 17 | 10 | Mediteraneen | N: 45°4'13' |
|  |  |  |  |  | females | 13 | 10 |  | E : 7°41'12'' |
| Spain | Barcelona | July | 2013 | 9 | females | 22 | 10 | Mediteraneen | N: 41°23'6.2' |
|  |  |  |  |  |  |  |  |  | E : 2°10'24'' |
| USA | Atlanta | October | 2018 | 33 | males | 25 | 10 | Humid Subtropical | N : 33°44'56'' |
|  |  |  |  |  | females | 24 | 10 |  | O : 84°23'16'' |
| Madagascar | Diego Suarez | NA | 2009 | >105 | males | 18 | - | Humid Tropical < 900m | S : 12°16'51.24'' |
|  |  |  |  |  | females | 14 | 10 |  | E : 49°17'27.092'' |
|  | Toamasina | December | 2010 | >105 | males | 22 | 10 | Humid Tropical < 900m | S : 18°8'59.64'' |
|  |  |  |  |  | females | 19 | 10 |  | E : 49°24'8.312' |
|  | Tsimbaza-Park | December | 2010 | >105 | males | 13 | 10 | Tropical > 900m | S : 18°55'40.395'' |
|  |  |  |  |  | females | 25 | 10 |  | E : 47°31'38.5' |
| Thailand | Chiang Mai | February | 2019 | Native | males | 8 | 8 | Tropical | N : 18° 42’ 46.1" |
|  |  |  |  |  |  |  |  |  | E : 98° 56’ 17.1" |
| Vietnam | Hô-chi-minh | October | 2012 | Native | females | 20 | 10 | Tropical | N : 10°47'19'' |
|  |  |  |  |  |  |  |  |  | E : 106°42'19'' |
|  | Binh Duong | October | 2012 | Native | females | 19 | 10 | Tropical | N : 10°47'19'' |
|  |  |  |  |  |  |  |  |  | E : 106°42'19' |
|  | Vüng Tau | October | 2012 | Native | males | 25 | 10 | Tropical | N : 10°22'26'' |
|  |  |  |  |  |  |  |  |  | E : 107°4'13'' |

**Table S2. Number and details on mosquito prevalence from this study and the previously published studies.**

| **Country** | **Site** | **Sex** | **Population age** | **Latitude (N)** | **Longitude (E)** | **Sample size** | **Uninfected** | **Infected** | **Prevalence** | **From** |
| --- | --- | --- | --- | --- | --- | --- | --- | --- | --- | --- |
| France | Feyzin | Female | 1 | 45.6736111 | 4.85916666666666 | 22 | 22 | 0 | 0,00 | This study |
| France | Feyzin | Male | 1 | 45.6736111 | 4.85916666666666 | 18 | 18 | 0 | 0,00 | This study |
| France | Nice | Female | 7 | 43.7 | 7.29249999999999 | 25 | 3 | 22 | 0,88 | This study |
| France | Nice | Male | 7 | 43.7 | 7.29249999999999 | 16 | 2 | 14 | 0,88 | This study |
| France | Oullins | Female | 1 | 45.7116667 | 4.80055555555555 | 18 | 16 | 2 | 0,11 | This study |
| France | Oullins | Male | 1 | 45.7116667 | 4.80055555555555 | 20 | 19 | 1 | 0,05 | This study |
| France | Portes-lès-Valence | Female | 2 | 44.8688889 | 4.86916666666666 | 31 | 24 | 7 | 0,23 | This study |
| France | Portes-lès-Valence | Male | 2 | 44.8688889 | 4.86916666666666 | 7 | 2 | 5 | 0,71 | This study |
| France | Saint Priest 2 | Male | 3 | 45.7188889 | 4.91916666666666 | 24 | 12 | 12 | 0,50 | This study |
| France | Saint Priest 1 | Female | 0 | 45.6858333 | 4.98055555555555 | 28 | 27 | 1 | 0,04 | This study |
| France | Saint Priest 1 | Male | 0 | 45.6858333 | 4.98055555555555 | 8 | 6 | 2 | 0,25 | This study |
| France | Saint Priest 2 | Female | 3 | 45.7188889 | 4.91916666666666 | 28 | 23 | 5 | 0,18 | This study |
| France | Villeurbanne | Female | 5 | 45.7711111 | 4.88888888888888 | 30 | 6 | 24 | 0,80 | This study |
| France | Villeurbanne | Male | 5 | 45.7188889 | 4.98055555555555 | 26 | 13 | 13 | 0,50 | This study |
| Italy | Torino | Female | 15 | 45.0702778 | 7.68666666666666 | 13 | 1 | 12 | 0,92 | This study |
| Italy | Torino | Male | 15 | 45.0702778 | 7.68666666666666 | 17 | 0 | 17 | 1,00 | This study |
| Madagascar | Diego-Suarez | Female | >105 | -12.2808333 | 49.2908333333333 | 18 | 1 | 17 | 0,94 | This study |
| Madagascar | Diego-Suarez | Male | >105 | -12.2808333 | 49.2908333333333 | 14 | 0 | 14 | 1,00 | This study |
| Madagascar | Tamatave | Male | >105 | -18.1497222 | 49.4022222222222 | 22 | 9 | 13 | 0,59 | This study |
| Madagascar | Tamatave | Female | >105 | -18.1497222 | 49.4022222222222 | 19 | 7 | 12 | 0,63 | This study |
| Madagascar | Tsimbazaza | Male | >105 | -18.9277778 | 47.5272222222222 | 13 | 2 | 11 | 0,85 | This study |
| Madagascar | Tsimbazaza | Female | >105 | -18.9277778 | 47.5272222222222 | 25 | 4 | 21 | 0,84 | This study |
| Spain | Barcelona | Female | 9 | 41.385 | 2.17333333333333 | 22 | 2 | 20 | 0,91 | This study |
| USA | Atlanta | Female | 33 | 33.7488889 | -84.38777777777779 | 24 | 6 | 18 | 0,75 | This study |
| USA | Atlanta | Male | 33 | 33.7488889 | -84.38777777777779 | 25 | 10 | 15 | 0,60 | This study |
| Vietnam | Binh Duong | Female | Native | 10.7886111 | 106.705277777777 | 19 | 7 | 12 | 0,63 | This study |
| Vietnam | Ho Chi Minh City | Female | Native | 10.7886111 | 106.705277777777 | 20 | 9 | 11 | 0,55 | This study |
| Vietnam | Vung Tau | Female | Native | 10.3738889 | 107.070277777777 | 25 | 4 | 21 | 0,84 | This study |
| Thailand | Phuket | Male | Native | 7.8788889 | 98.3983333333333 | 21 | 11 | 10 | 0,48 | This study |
| France | Feyzin | Female | 1 | 45.6736111 | 4.85916666666666 | 22 | 0 | 22 | 1,00 | This study |
| France | Feyzin | Male | 1 | 45.6736111 | 4.85916666666666 | 18 | 0 | 18 | 1,00 | This study |
| France | Nice | Female | 7 | 43.7 | 7.29249999999999 | 25 | 22 | 3 | 0,12 | This study |
| France | Nice | Male | 7 | 43.7 | 7.29249999999999 | 16 | 14 | 2 | 0,13 | This study |
| France | Oullins | Female | 1 | 45.7116667 | 4.80055555555555 | 18 | 2 | 16 | 0,89 | This study |
| France | Oullins | Male | 1 | 45.7116667 | 4.80055555555555 | 20 | 1 | 19 | 0,95 | This study |
| France | Portes-lès-Valence | Female | 2 | 44.8688889 | 4.86916666666666 | 31 | 7 | 24 | 0,77 | This study |
| France | Portes-lès-Valence | Male | 2 | 44.8688889 | 4.86916666666666 | 7 | 5 | 2 | 0,29 | This study |
| France | Saint Priest 2 | Male | 3 | 45.7188889 | 4.91916666666666 | 24 | 12 | 12 | 0,50 | This study |
| France | Saint Priest 1 | Female | 0 | 45.6969444 | 4.98055555555555 | 28 | 1 | 27 | 0,96 | This study |
| France | Saint Priest 1 | Male | 0 | 45.6969444 | 4.98055555555555 | 8 | 2 | 6 | 0,75 | This study |
| France | Saint Priest 2 | Female | 3 | 45.7188889 | 4.91916666666666 | 28 | 5 | 23 | 0,82 | This study |
| France | Villeurbanne | Female | 5 | 45.7711111 | 4.88888888888888 | 30 | 24 | 6 | 0,20 | This study |
| France | Villeurbanne | Male | 5 | 45.7188889 | 4.91916666666666 | 26 | 13 | 13 | 0,50 | This study |
| Italy | Torino | Female | 15 | 45.0702778 | 7.68666666666666 | 13 | 12 | 1 | 0,08 | This study |
| Italy | Torino | Male | 15 | 45.0702778 | 7.68666666666666 | 17 | 17 | 0 | 0,00 | This study |
| Madagascar | Diego-Suarez | Female | 105 | -12.2808333 | 49.2908333333333 | 18 | 17 | 1 | 0,06 | This study |
| Madagascar | Diego-Suarez | Male | 105 | -12.2808333 | 49.2908333333333 | 14 | 14 | 0 | 0,00 | This study |
| Madagascar | Tamatave | Male | 105 | -18.1497222 | 49.4022222222222 | 22 | 13 | 9 | 0,41 | This study |
| Madagascar | Tamatave | Female | 105 | -18.1497222 | 49.4022222222222 | 19 | 12 | 7 | 0,37 | This study |
| Madagascar | Tsimbazaza | Male | 105 | -18.9277778 | 47.5272222222222 | 13 | 11 | 2 | 0,15 | This study |
| Madagascar | Tsimbazaza | Female | 105 | -18.9277778 | 47.5272222222222 | 25 | 21 | 4 | 0,16 | This study |
| Spain | Barcelona | Female | 9 | 41.385 | 2.17333333333333 | 22 | 20 | 2 | 0,09 | This study |
| USA | Atlanta | Female | 33 | 33.7488889 | -84.38777777777779 | 24 | 18 | 6 | 0,25 | This study |
| USA | Atlanta | Male | 33 | 33.7488889 | -84.38777777777779 | 25 | 15 | 10 | 0,40 | This study |
| Vietnam | Binh Duong | Female | 200 | 10.7886111 | 106.705277777777 | 19 | 12 | 7 | 0,37 | This study |
| Vietnam | Ho Chi Minh City | Female | 200 | 10.7886111 | 106.705277777777 | 20 | 11 | 9 | 0,45 | This study |
| Vietnam | Vung Tau | Female | 200 | 10.3738889 | 107.070277777777 | 25 | 21 | 4 | 0,16 | This study |
| Thailand | Phuket | Male | 200 | 7.8788889 | 98.3983333333333 | 21 | 10 | 11 | 0,52 | This study |
| USA | East St Louis | Larvae | 14 | 38.616405 | -90.159821 | 55 | 0 | 55 | 1,00 | Aliabadi et al. 2002 |
| USA | Peoria_1 | Larvae | 2 | 40.703544 | -89.579084 | 49 | 14 | 35 | 0,71 | Aliabadi et al. 2002 |
| USA | Peoria_2 | Larvae | 1 | 40.703545 | -89.579086 | 52 | 43 | 9 | 0,17 | Aliabadi et al. 2002 |
| USA | Florida_1 | Larvae | 4 | 27.994401 | -81.760253 | 883 | 300 | 583 | 0,66 | Blackmore et al. 1995 |
| USA | Florida_2 | Larvae | 1 | 27.994402 | -81.760254 | 535 | 433 | 102 | 0,19 | Blackmore et al. 1995 |
| USA | Hawaï | Mixed | 117 | 19.896766 | -155.582782 | 118 | 19 | 99 | 0,84 | Seabourn et al. 2020 |
| USA | Orleans_Parish_1 | Female | 9 | -90,0715323 | 0,716049383 | 181 | 55 | 126 | 0,70 | Comiskey et al. 1999 |
| USA | Orleans_Parish_2 | Male | 9 | -90,0715323 | 0,716049383 | 62 | 14 | 48 | 0,77 | Comiskey et al. 2000 |

**Table S3. Fixed factors impact on the microbial divervisity associated with the Asian tiger mosquito.**

| **Class** | **Response Variable** | **Fixed Factors** | **Sex** | **pseudoF/χ²** | ***p-value*** | **R^2^** |
| --- | --- | --- | --- | --- | --- | --- |
| Eukaryotic microbiota | Richness (GLM) | Origin |  | 593.93 | **<0.0001***** |  |
|  |  | Sex |  | 4.01 | **0.0453*** |  |
|  |  | Origin x Sex |  | 181.48 | **<0.0001***** |  |
|  | Alpha diversity (LM) | Origin |  | 7.47 | **<0.0001***** |  |
|  |  | Sex |  | 2.81 | 0.0950 |  |
|  |  | Origin x Sex |  | 1.6457 | 0.1239 |  |
|  | Beta diversity (permANOVA) | Origin |  | 7.19 | **0.001 ***** | 0.32 |
|  |  | Sex |  | 1.17 | 0.28 | 0,00 |
|  |  | Origin x Sex |  | 1.81 | **0.001 ***** | 0.04 |
|  |  | Years after introduction | Female | 4.7368 | **0.001 ***** | 0.22 |
|  |  | Years after introduction | Male | 4.8806 | **0.001 ***** | 0.24 |
|  |  | Climate | Female | 3.8823 | **0.001 ***** | 0.08 |
|  |  | Climate | Male | 4.7886 | **0.001 ***** | 0.13 |
| Prokaryotic microbiota | Richness (GLM) | Origin |  | 867.33 | **<0.0001***** |  |
|  |  | Sex |  | 0.12 | 0.7319 |  |
|  |  | Origin x Sex |  | 162.12 | **<0.0001***** |  |
|  | Alpha diversity (LM) | Origin |  | 15.14 | **<0.0001***** |  |
|  |  | Sex |  | 0.78 | 0.38 |  |
|  |  | Origin x Sex |  | 19.46 | **0.0087**** |  |
|  | Beta diversity (permANOVA) | Origin |  | 7.65 | **0.001 ***** | 0.32 |
|  |  | Sex |  | 7.08 | **0.001 ***** | 0.02 |
|  |  | Origin x Sex |  | 3.57 | **0.001 ***** | 0.07 |
|  |  | Years after introduction | Female | 3.7418 | **0.001 ***** | 0.19 |
|  |  | Years after introduction | Male | 4.2692 | **0.001 ***** | 0.21 |
|  |  | Climate | Female | 5.6248 | **0.001 ***** | 0.11 |
|  |  | Climate | Male | 5.9576 | **0.001 ***** | 0.16 |

**
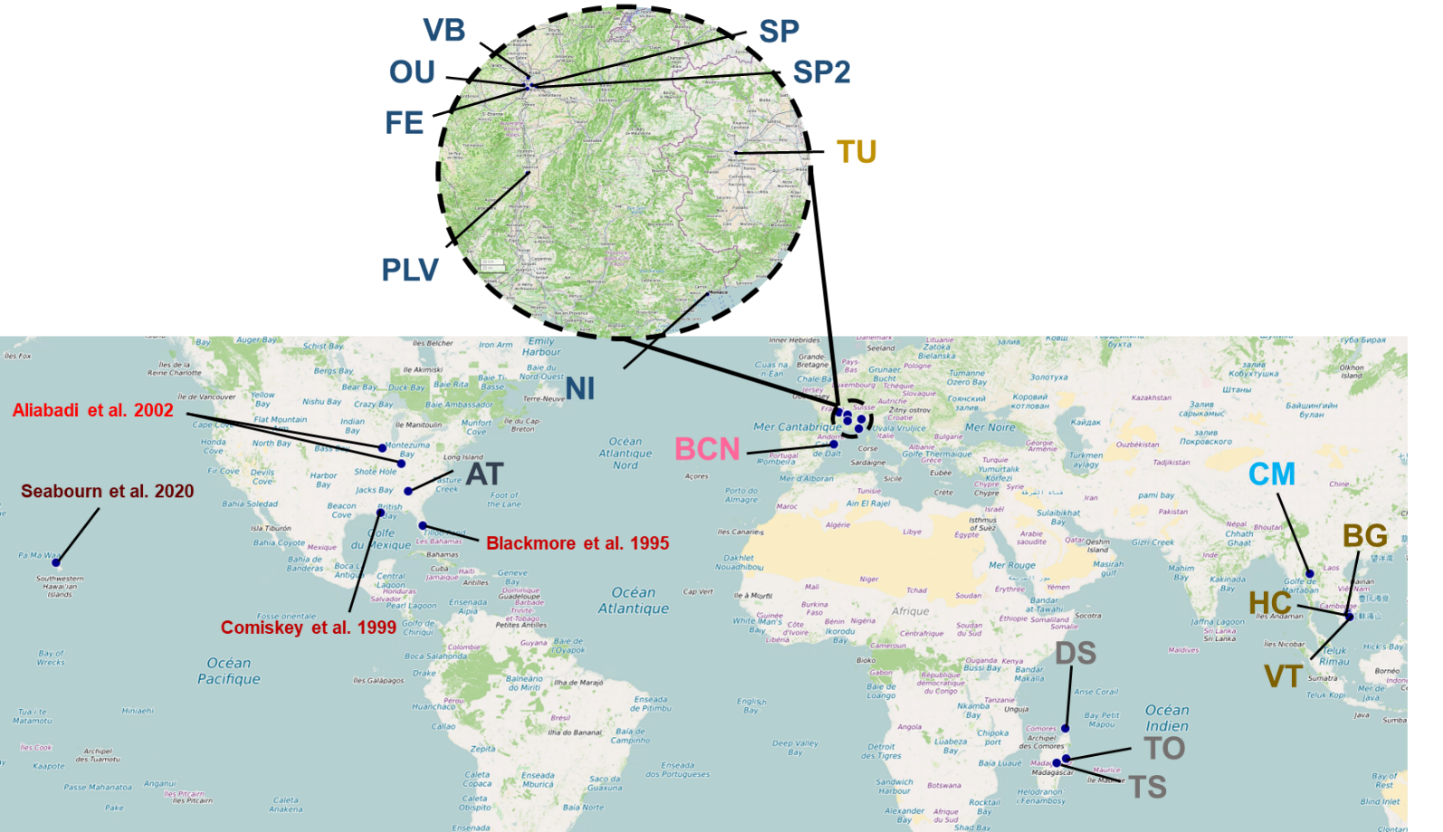
**

**Figure S1. World map representing the collection sites of mosquito populations used in this study and those for which data were extracted from previously published studies.** Each dot represents a sample site and the name of the localization has been reported with letters referring to the name of the city for data acquired in this study (FE = Feyzin, NI = Nice, OU = Oullins, PLV = Portes-lès-Valence, SP = Saint Priest, VB = Villeurbanne, TU = Torino, DS = Diego-Suarez, TO = Toamasina, TS = Tsimbaza, BCN = Barcelona, CM = Chiang Mai, BG = Binh Duong, HC = Hô-chi-minh, VT = Vüng Tau, AT = Atlanta) or the first authors and publication date for data collected from previously published studies.


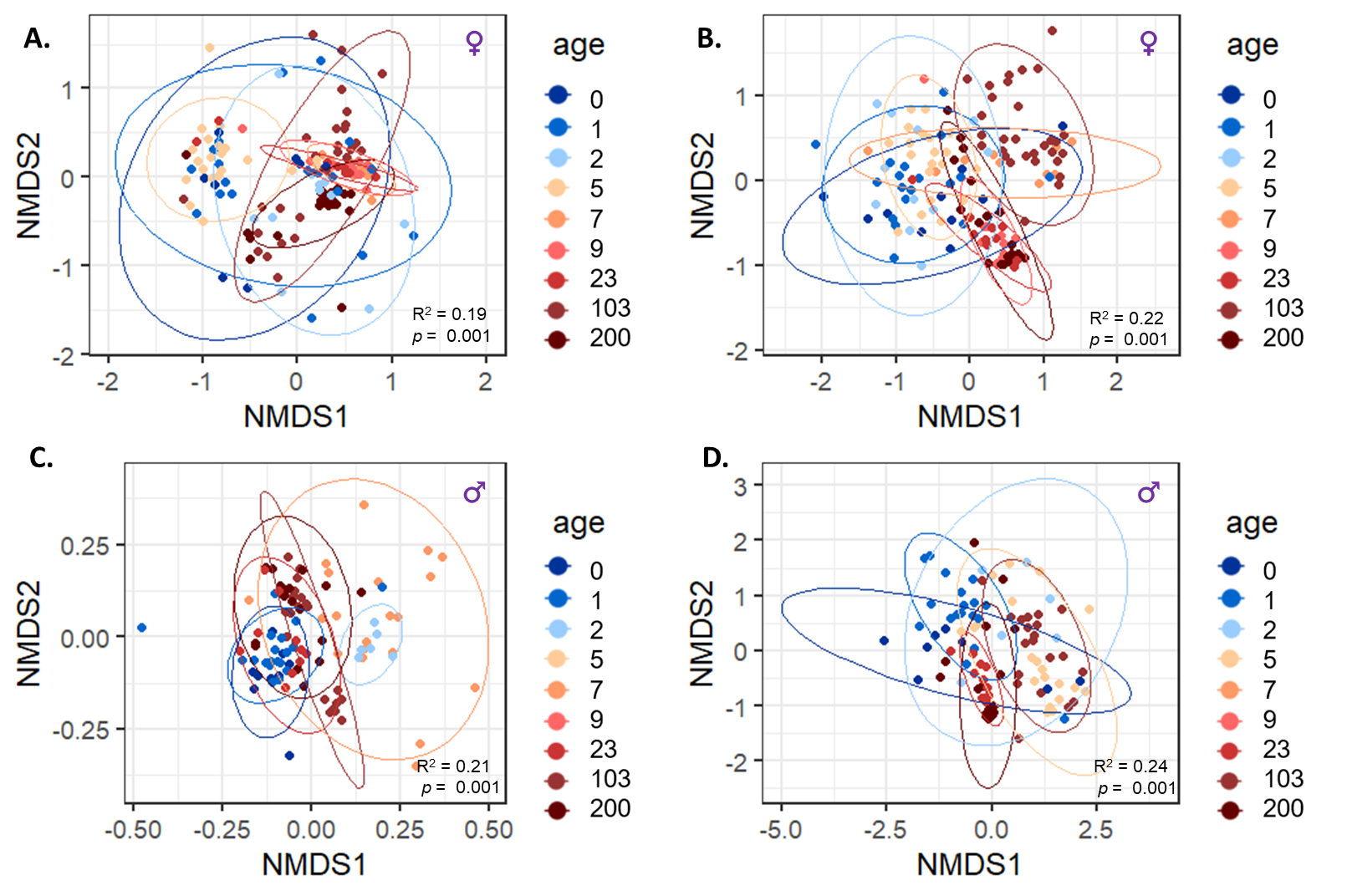


**Figure S2. Microbiota composition dissimilarity separation according to mosquito population ages.** For both (A, B) female and (C, D) male individuals the dissimilarity (bray-curtis index) of (A, C) prokaryotic and (B, D) eukaryotic microbiota communities was represented. The points are colored with a gradient ranging from blue to red based on the age of each population (years after introduction). Native populations were set to 200 years.


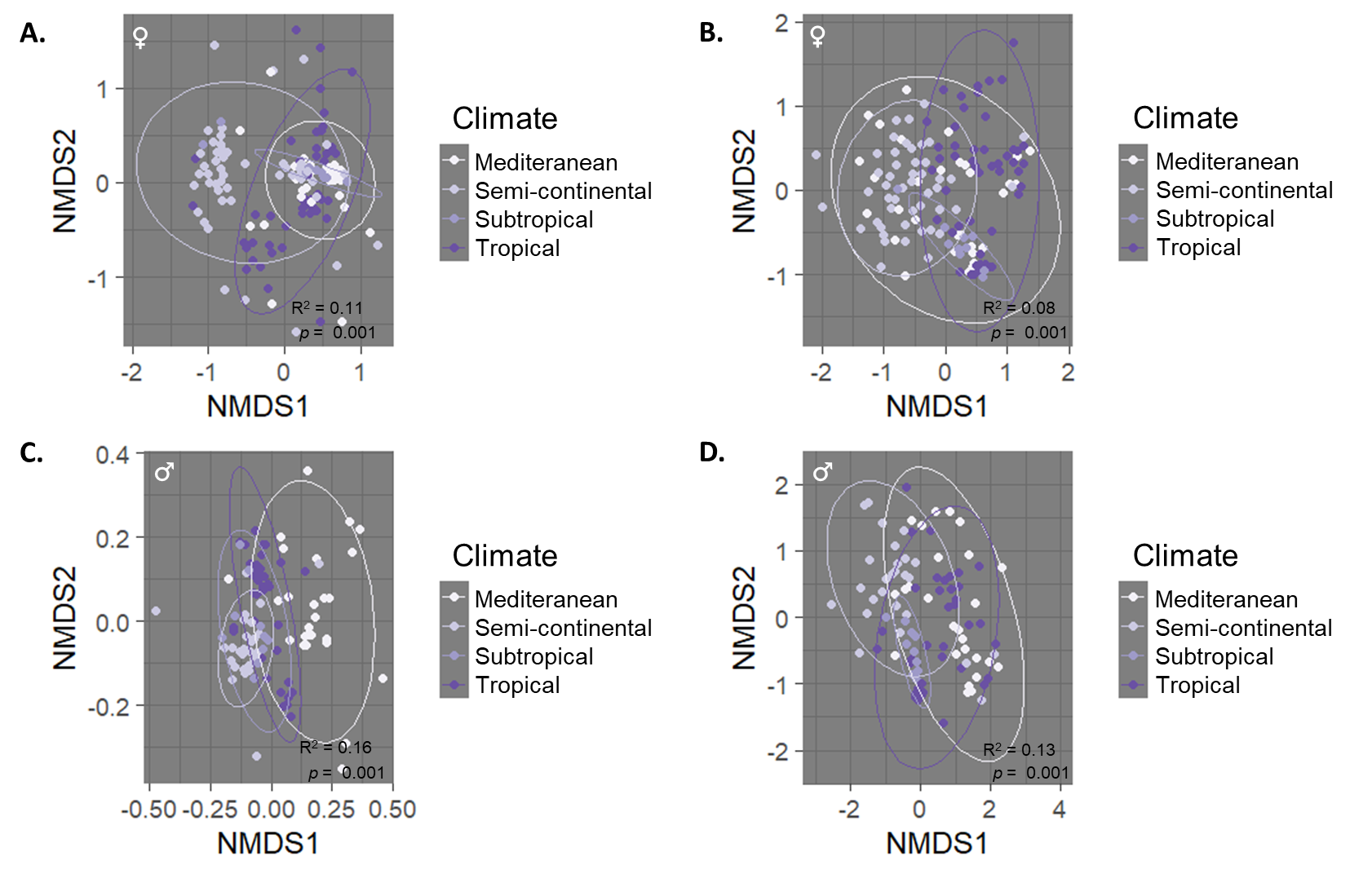


**Figure S3. Microbiota dissimilarity separation according to climate.** For both (A, B) female and (C, D) male individuals the dissimilarity (bray-curtis index) of (A, C) prokaryotic and (B, D) eukaryotic microbiota communities was represented. The points are colored with a gradient ranging from blue to red based on climatic regions. Native populations were set to 200 years.


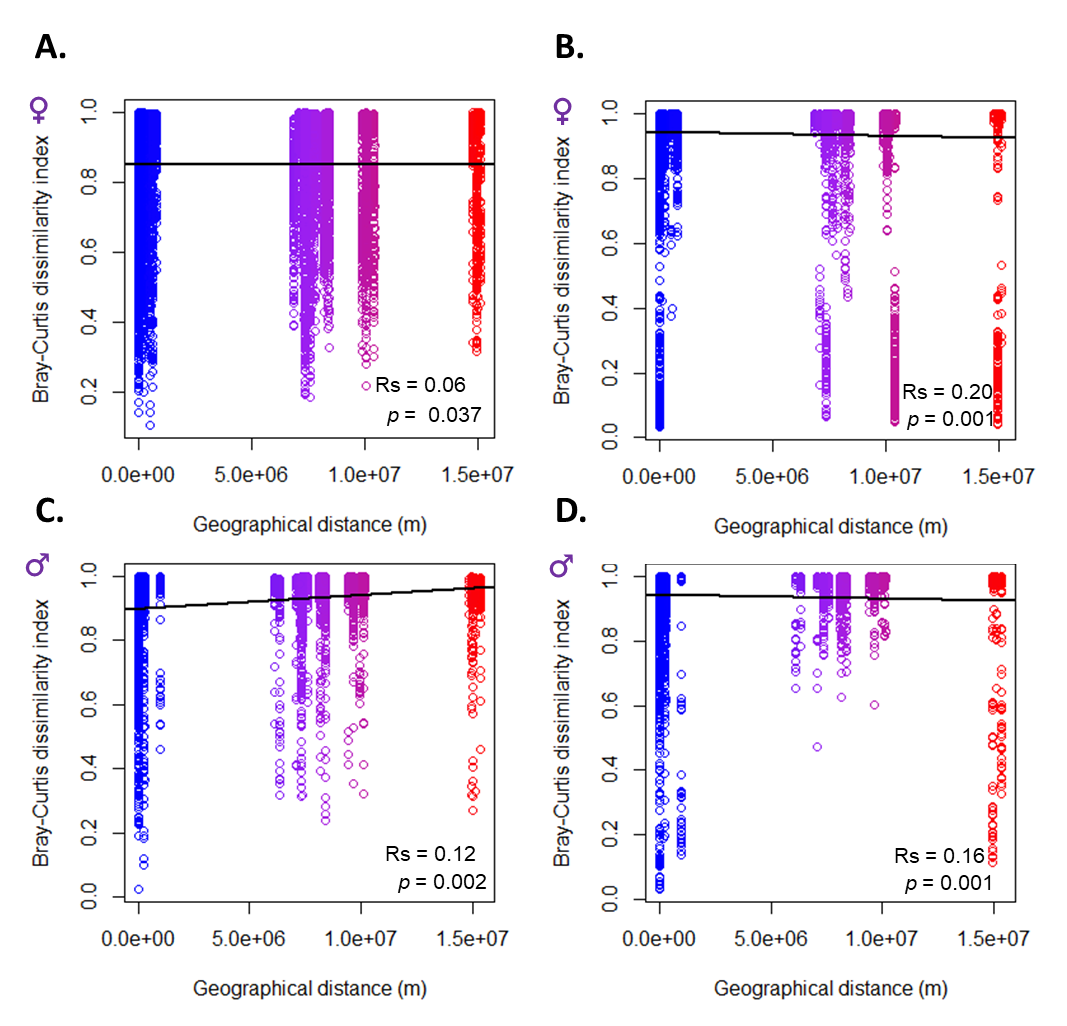


**Figure S4. Microbiota dissimilarity correlation with geographical distances.** Spearman correlation analysis between the microbiota community dissimilarity estimated with the Bray-Curtis index and geographical ellipsoid distance. Correlations were represented for both (A, B) female and (C, D) male individuals and for (A, C) prokaryotic and (B, D) eukaryotic microbiota communities. The points are colored with a gradient ranging from blue to red based on geographical distance between both points being compared. The correlation coefficient (Rs) and p-value are represented on each graph.


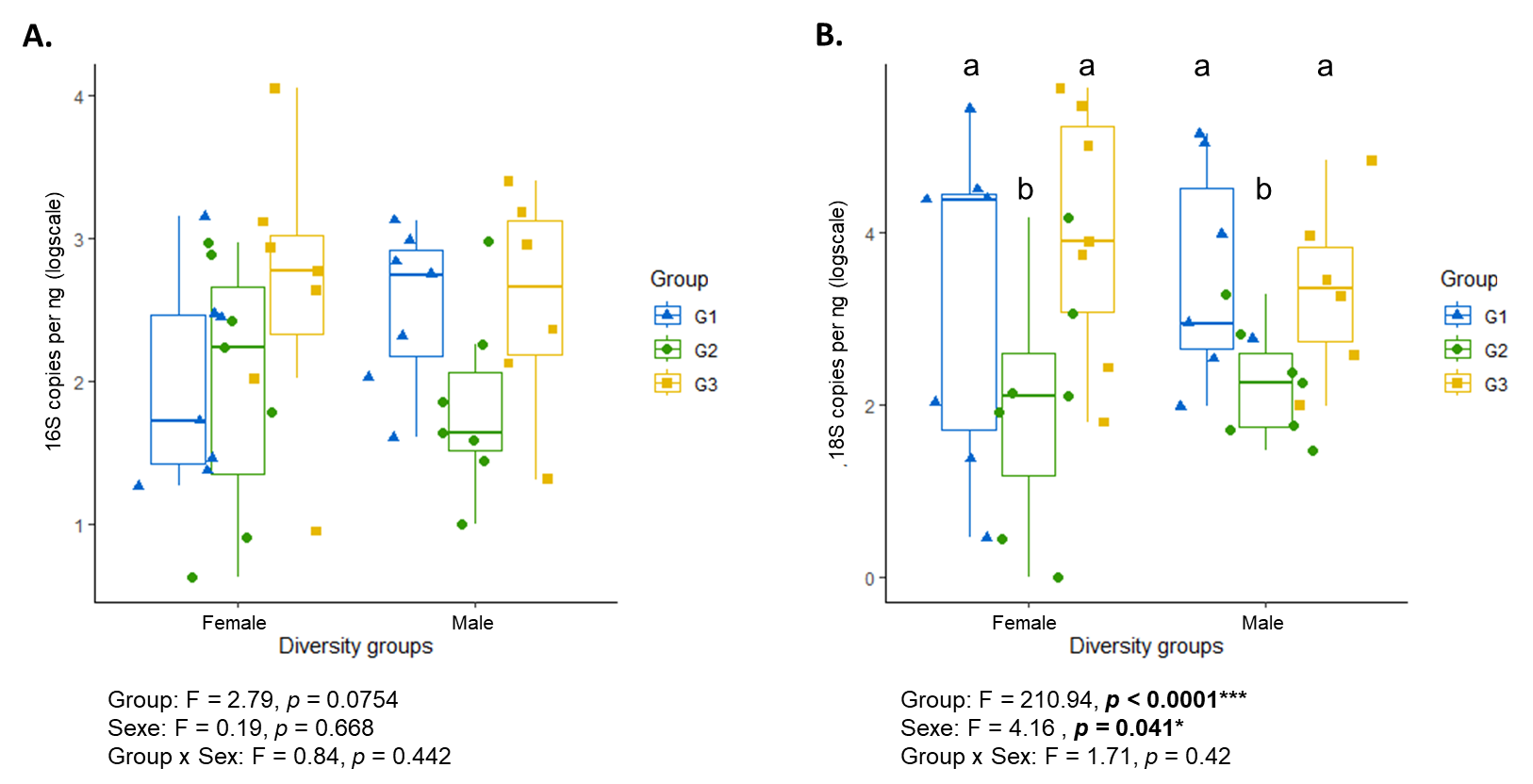


**Figure S5. Quantification of microbial densities.** Quantifications were performed through qPCR of the (A) 16S rDNA genes for the eukaryotic community or (B) 18S rDNA gene for the prokaryotic community. Females and males are respectively colored in red and blue. G1 represent samples from various origins for which the *Ascogregarina taiwanensis* Otu000001 dominates the microbiota. G2 represents samples for which *As. taiwanensis* Otu000002 and Otu000003 dominates the microbiota. Finally, G3 represents samples that are not dominated by *As. taiwanensis*.
